# Human peripheral osteoclast-precursor-development patterns reveal the significance of RPS17-dependent ribosome synthesis to Ankylosing Spondylitis lesions

**DOI:** 10.1038/s41413-025-00474-5

**Published:** 2025-12-04

**Authors:** Dianshan Ke, Hanhao Dai, Junyong Han, Yibin Su, Hongyi Zhu, Rongsheng Zhang, Tingwei Gao, Linhai Yang, Yunlong Yu, Xiaochun Bai, Changqing Zhang, Jie Xu

**Affiliations:** 1 High-Level Research Platform for Fujian Provincial Medical Creating Double-High Project, Department of Orthopedics, Fuzhou University Affiliated Provincial Hospital, Fujian Provincial Hospital, Shengli Clinical Medical College of Fujian Medical University, Fuzhou, China; 2 Institute of Basic Medicine, School of Basic Medical Sciences, Fujian Medical University, Fuzhou, China; 3 Academy of Orthopedics, Guangdong Province, Guangdong Provincial Key Laboratory of Bone and Joint Degeneration Diseases, The Third Affiliated Hospital of Southern Medical University, Guangzhou, Guangdong China; 4 Institute for Immunology, Fujian Academy of Medical Sciences, Fuzhou, China; 5 Institute of Microsurgery on Extremities, Department of Orthopaedics, Shanghai Sixth People’s Hospital Affiliated to Shanghai Jiao Tong University School of Medicine, Shanghai, China

**Keywords:** Bone, Pathogenesis

## Abstract

Osteoclast-development patterns and their alterations across Ankylosing Spondylitis (AS) conditions are mysterious, making AS treatment difficult. Our study aims to clarify osteoclast-precursor (OCP) development patterns from monocytes and their variations under AS conditions. We performed single-cell transcriptomics in peripheral blood mononuclear cells (PBMCs) from healthy donors and AS patients in the early, aggravated and remission stages. After monocytic reclustering, OCP-development patterns and the alterations upon AS onset and different outcomes were revealed based on single-cell trajectory. The trajectories revealed two monocyte states with strong OCP features, and AS pathogenesis was characterized by their reduction. Ribosome synthesis was considered the essential function for the development towards OCP-featured states, and this function and its representative molecule, RPS17, showed a decreasing trend with AS onset and outcomes. Histology assessment showed that RPS17 underexpression participated in AS inflammatory osteogenesis and ankylosing destruction. Conditional knockout of RPS17 ameliorated ovariectomy-induced bone loss and enhanced osteoclastogenesis, and RPS17 overexpression improved the phenotype of AS-like mice. Importantly, local injection of RPS17-overexpressed monocytic OCPs markedly ameliorated the joint alterations of AS-like mice without promoting bone loss; this was associated with enhanced osteoclastogenesis adjacent to the articular surface and T-cell-suppressive property in monocytic OCPs. Overall, the evolution of monocytes towards OCP-lineage fate mainly depends on ribosome synthesis, and OCP-development disorder participates in AS lesions due to a reduction in RPS17-dependent ribosome synthesis. Notably, RPS17-overexpressed monocytic OCPs have translational potential in preventing and treating AS peripheral lesions.

## Introduction

Ankylosing Spondylitis (AS) is a chronic rheumatic disease that mainly involves sacroiliac joints, spine and peripheral joints.^1,2^ Typical cases of AS can manifest as progressive erosion and fusion of articular cartilage, ultimately leading to ankylosing deformities in the spine and joints; these deformities are the main factors causing disability.^3,4^ Nevertheless, AS pathogenesis is still unclear. Aberrant osteogenesis under inflammatory conditions is the pathological basis for joint fusion and ankylosis; its treatment remains an insurmountable challenge. Current therapy focuses on symptom relief and disease improvement with drugs such as nonsteroidal drugs (NSAIDs), conventional synthetic disease-modifying antirheumatic drugs (csDMARDs) and biological DMARDs (bDMARDs) inhibiting tumor necrosis factor (TNF) and interleukin-17 (IL-17).^5,6^ At present, there are no other effective strategies besides surgical procedures to deal with late-stage disabled patients. Revealing the detailed mechanism underlying AS inflammatory osteogenesis is crucial for improving its treatment.

At present, multiple AS studies focus on osteoblast-dependent abnormal osteogenesis.^7–9^ However, the factors leading to inflammatory osteogenesis are complex under AS conditions, suggesting that there are other pathological reasons besides excessive osteogenesis. The dynamic balance between osteogenesis and bone resorption maintains normal bone remodeling. Defective osteoclastogenic ability can result in increased bone mass, thereby causing osteopetrosis.^10,11^ AS is characterized by the coexistence of focal new-bone formation and systemic bone loss. Abundant studies support that enhanced osteoclastogenesis leads to AS-related bone loss.^8,12–14^ However, local hyper-osteogenesis requires support from a reduction in osteoclastogenic capacity. We speculate that AS patients may experience locally damaged osteoclastogenesis; this is hinted by some studies.^15,16^ Circulating monocytes, significant immunocytes involved in AS lesions,^17^ are considered peripheral OCPs with strong osteoclastogenic ability.^18,19^ Monocytes from AS patients are weak in osteoclastogenesis and their capacities are negatively correlated with disease duration,^20^ suggesting the possible significance of peripheral OCPs for inflammatory osteogenesis. However, osteoclastogenic research associated with AS is currently scarce. The innovation of our study is that it relies on AS-associated osteoclastogenic behavior to explore the underlying mechanism of AS inflammatory osteogenesis from a bone-resorption perspective.

To achieve our goals, we need to have a clear understanding for osteoclastogenic patterns of peripheral OCPs. Here, we adopted a novel approach: based on PBMCs-associated single-cell RNA sequencing (scRNA-seq), our monocytic reclusterings presented cytotaxonomic hallmarks in monocytes linked to AS onset (early active stage), aggravation (late disabled stage) and clinical remission, and human OCP-development patterns in the periphery were revealed relying on single-cell trajectory, including the development route of monocytes towards OCP-lineage fate and responsible genes and functions. On these foundations, the alterations upon AS onset and different outcomes were clarified; this provides important clues for AS inflammatory osteogenesis.

## Results

### Single-cell transcriptional profiling of monocytes

**Fig. 1:**
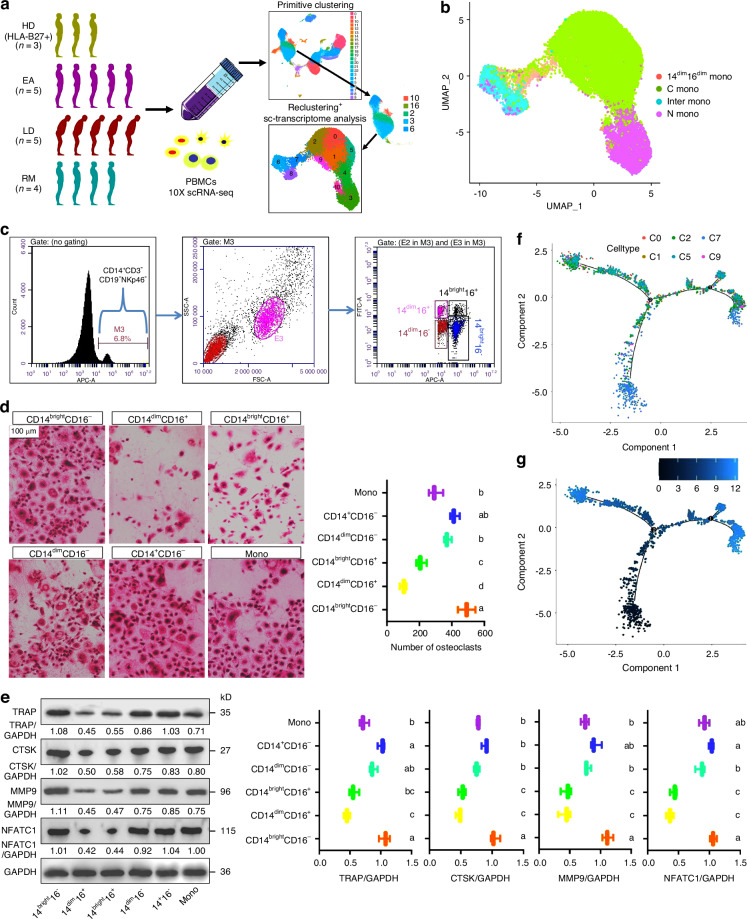
Single-monocyte transcriptional profiling and identification of monocytic OCPs. **a** Schematic diagram showing the overall study design. The scRNA-seq was based on PBMCs across four conditions and the output data were applied for sc-transcriptome analysis and primitive clustering. Five out of 23 clusters that fit the genetic characteristics of monocytes were reclustered and subjected to sc-transcriptome analysis again. **b** UMAP projection based on monocytic types, and each dot is colored according to cell subtype. **c** Gating strategy for monocyte enrichment and clustering. Monocytes (CD14^+^CD3^−^CD19^−^NKp46^−^ cells) were enriched from PBMCs, and four specific monocyte populations were sorted from the CD14^+^ gate based on CD14 and CD16 expression using FACS. **d** Osteoclastogenic capacities of various monocytic clusters (CD14^bright^CD16^−^, CD14^dim^CD16^+^, CD14^bright^CD16^+^, CD14^dim^CD16^−^ and CD14^+^CD16^−^ cells) and total monocytes. Scale bar: 100 µm. **e** Western-blotting analysis of osteoclastic markers (TRAP, CTSK, MMP9 and NFATC1) among five monocytic clusters and total monocytes. The relative levels of each protein are expressed as the ratio of the target protein to GAPDH. **f** Merged cell-trajectories of mono subsets 0/1/2/5/7/9 (Monocle-2). Different dots correspond to different subsets. **g** Pseudotimeline of cell-trajectories. The deeper dot color indicates a smaller pseudotime, i.e., an earlier development stage. The changes from a to b/from b to c/from c to d indicate a significant decrease with *P* < 0.05, and double-letter indicates no statistical difference between the given group and the compared groups marked with a single letter

To characterize monocytic immune hallmarks of AS patients, we performed a scRNA-seq on PBMCs from 14 patients and 3 healthy donors (HDs) (Fig. 1a and Table S1). AS patients were divided into three groups based on clinical features, laboratory findings and imaging results: untreated first-time patients with high activity (EAs); patients with spinal deformities or peripheral joint destruction (LDs); and patients with clinical remission after standardized treatment (RMs). Relying on Seurat-based clustering of uniform manifold approximation and projection (UMAP), we captured transcripts of 23 cell clusters (Fig. 1a). Clusters-2/3/6/10/16 were defined as monocytes according to their gene-expression characteristics, and were integrated for transcriptome analysis (Figs. 1a and S1a). Notably, the inclusion of cluster-16 is due to the obvious expression of LYZ and TYROBP and the scarcity of other markers, while the exclusion of cluster-21 is due to their free state observed through UMAP and closer proximity to a rare type of dendritic cells (DCs), double-negative DCs,^21^ with a lack of *CD1C* and *THBD* and high expression of *CSF3R* and *NEAT1* (Fig. S1a). After reclustering, 38 654 monocytes were divided into 11 subsets with respective gene-distribution characteristics (Fig. S1b–d). Based on CD14/CD16 expression status, subsets-0/1/2/5/9 were annotated as classic monocytes (C monos; CD14^bright^CD16^dim^), subsets-3/10 were characterized by nonclassic monocytes (N monos; CD14^dim^CD16^bright^), subsets-4/6/8 were annotated as intermediate monocytes (inter monos; CD14^bright^CD16^bright^), and subset-7 tended to be 14^dim^16^dim^ monos due to the absence of *CD14* and *CD16* (Figs. 1b and S1e), which are reported previously.^22^ The proportion of C monos to total monocytes in EAs was higher than that in HDs (Fig. S1f, g), similar to previous reports demonstrating an increase in C mono abundance in AS/AS/spondyloarthritis (SpA) patients.^17^ GSVA showed that the other three monocyte-subtypes had their preferred functions while 14^dim^16^dim^ monos were more involved in multinuclear osteoclast differentiation, osteoclast fusion and mucosal innate immune response (Fig. S1h).

### Osteoclastogenic potential of different monocytic types

Then, osteoclastogenic potential of different monocyte subtypes was compared. After enriching different monocyte-clusters with corresponding strategies (Fig. 1c), we discovered that osteoclasts derived from CD14^dim^CD16^−^ (representing 14^dim^16^dim^ monos) and CD14^bright^CD16^−^ (representing C monos) cells far exceeded those of other two clusters (representing N monos and inter monos), and those of CD14^bright^CD16^−^ cells were the most abundant (Fig. 1d). Western blotting described similar trends, showing that CD14^dim^CD16^−^ and CD14^bright^CD16^−^ cells had higher protein levels in CTSK, MMP9, TRAP and NFATC1 (Fig. 1e). The strong osteoclastogenic ability of C monos conformed to previous studies.^18,19,23,24^ Due to negligible expression of two markers, 14^dim^16^dim^ monos have obvious pluripotency, which manifests as their preference for fusion and multinucleation (Fig. S1h); this explains to some extent their osteoclastogenic capacity. We accordingly believe that CD16^dim^ monos (similar to the sum of CD14^dim^CD16^−^ cells and CD14^bright^CD16^−^ cells, i.e., CD14^+^CD16^−^ monocytes) act as appropriate monocytic precursors for osteoclastogenesis, i.e., monocytic OCPs; this conforms to previous research.^18,19^ Cell trajectory analysis of CD16^dim^ monocytes revealed that most subset-7 cells were in the early stage of pseudotimeline (Figs. 1f, g and S2); this conforms to our inference, supporting the pluripotency of 14^dim^16^dim^ monos.

### AS is characterized by a reduction in monocytic evolution to OCP-lineage fate

**Fig. 2:**
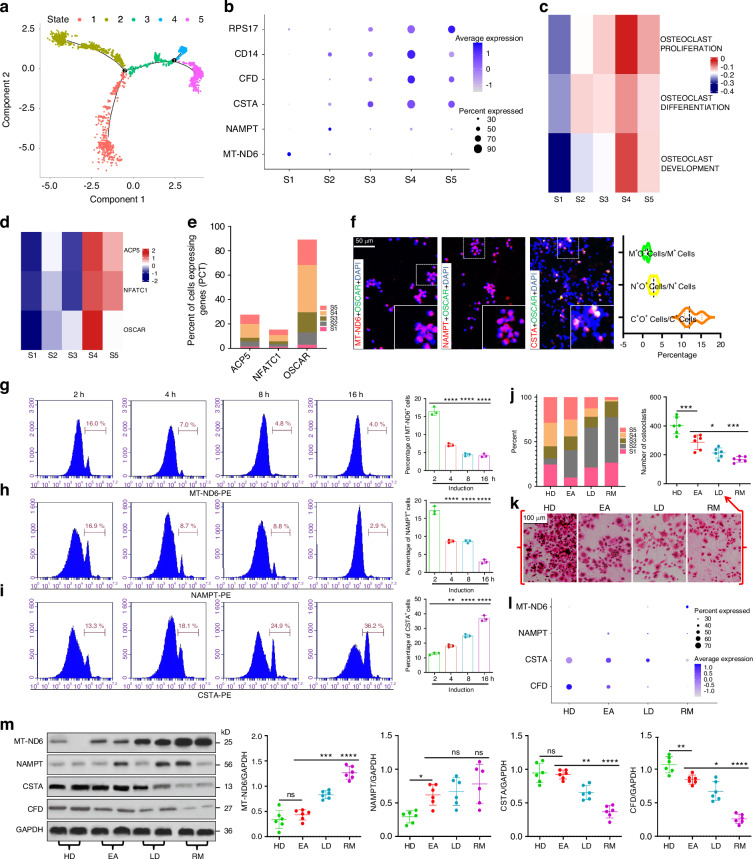
AS-associated monocytic evolution towards OCP-lineage fate. **a** Merged cell-trajectories of the above six subsets showing 5 cell-states and 2 cell-branches. State-1 is the starting point for differentiation. **b** Bubble plots showing the expression distribution of top genes belonging to various cell-states across five states. Circle size indicates percentage of cells expressing corresponding gene (PCT) and color depth indicates average expression levels. **c** GSVA scores in the osteoclastogenic functions between five states. Rows were normalized, and the transition of blue-white-red indicates an increase in the scores. **d** Heatmap showing relative intergroup comparisons of *ACP5*, *NFATC1* and *OSCAR* expression between five states. Rows were normalized, and the transition of blue-white-red indicates an increase in the gene expression. **e** PCT of *ACP5*, *NFATC1* and *OSCAR* across five states. **f** Immunofluorescence analysis of co-expression of MT-ND6, NAMPT and CSTA with OSCAR in CD14^+^CD16^−^ monocytes. M^+^O^+^ Cells/M^+^ Cells represents the ratio of MT-ND6 and OSCAR-double-positive cells to MT-ND6-positive cells; N^+^O^+^ Cells/N^+^ Cells represents the ratio of NAMPT and OSCAR-double-positive cells to NAMPT-positive cells; C^+^O^+^ Cells/C^+^ Cells represents the ratio of CSTA and OSCAR-double-positive cells to CSTA-positive cells. **g**–**i** Flow cytometry of CD14^+^CD16^−^ monocytes identifying the approximate trend of MT-ND6^+^, NAMPT^+^ and CSTA^+^ cells at the 2nd, 4th, 8th and 16th hours of osteoclastic induction. **j** Proportions of five states from four groups. **k** Osteoclast differentiation capacities for CD14^+^CD16^−^ monocytes of each three subjects from four groups. Scale bar: 100 µm. Data come from the mean values of osteoclasts in each three wells. **l** The expression distribution of MT-ND6, NAMPT, CSTA and CFD across four groups. Circle size indicates PCT and color depth indicates average levels. **m** Western-blotting analysis of MT-ND6, NAMPT, CSTA and CFD for CD14^+^CD16^−^ monocytes from four groups of subjects (*n* = 6/group; each 2 samples presented in one group). *P < 0.05, **P < 0.01, ***P < 0.001, ****P < 0.000 1 and ns (not significant) by one-way ANOVA

As pseudotimeline progressed, cell distribution was divided into five states (Fig. 2a). There were two branches involved in cell development (Fig.< span Class="cite" data-de-type="Figure" data-rid="Fig2" href="f2" ID="MPS_d1e381" Name="fig">2a). State-1, mainly composed of 14^dim^16^dim^ monos, conforms to the characteristics of pre-monocytes. State-1 developed into states-2/3 in branch 2, and state-3 developed into states-4/5 in branch 1 (Fig. 2a). The top genes of five cell-states were as follows: *MT-ND6* for state-1, *NAMPT* for state-2, *CSTA* for states-3/4/5, *CFD* and *CD14* for state-4, and *RPS17* for states-4/5 (Fig. 2b). In terms of top-gene expression, states-1/2 exhibited significant heterogeneity, whereas the other states had similarities. States-4/5 had high osteoclastogenic values (osteoclast development/differentiation/proliferation) and strong expression of osteoclastic genes (*ACP5*, *NFATC1*, *OSCAR*), with state-4 ranking first (Fig. 2c–e). Therefore, we speculate that the progression of state-1 to state-3 is more inclined to the development of monocytes towards osteoclasts, and using state-3 as the starting point, state-4 was closer to advanced OCPs. Correlation analysis revealed the role of cell abundance of states-3/4/5 in adjusting development direction towards OCP-lineage fate (Fig. S3a, b), suggesting the credibility of the above trajectories. As expected, after enriching CD14^+^CD16^−^ monocytes, CSTA-positive cells showed strong OSCAR fluorescence, while that in MT-ND6- or NAMPT-positive cells was dim (Figs. 2f and S3c). We relied on osteoclastic induction to identify OCP-development dynamics. With osteoclastic induction, MT-ND6- or NAMPT-positive cells gradually decreased while CFD-positive cells gradually increased (Fig. 2g–i); this is important evidence for cell-development trajectory. Additionally, cells in state-2 gradually increased from HDs to LDs/RMs, while cells in states-4/5 gradually decreased (Figs. 2j and S3d). Therefore, we infer that decreased cells in states-4/5 participates in AS onset and outcomes. Osteoclasts from CD14^+^CD16^−^ monocytes in AS groups were less than those in HDs, but those in EAs were the most abundant among AS groups (Fig. 2k), indicating that peripheral osteoclastogenesis decreases upon AS onset and outcomes. Moreover, MT-ND6 showed an overall increasing trend from HDs to LDs/RMs and NAMPT was overexpressed in AS groups, whereas CSTA and CFD gradually decreased (Fig. 2l); the above trend was verified by protein detection, showing that MT-ND6 expression gradually increased from HDs to LDs/RMs and NAMPT expression was higher in AS groups, while CSTA and CFD displayed a completely opposite trend (Fig. 2m). These results unmasked the consistency between OCP-development potential and osteoclastogenic capacity across AS conditions.

### Ribosome synthesis contributes to peripheral OCP development

**Fig. 3:**
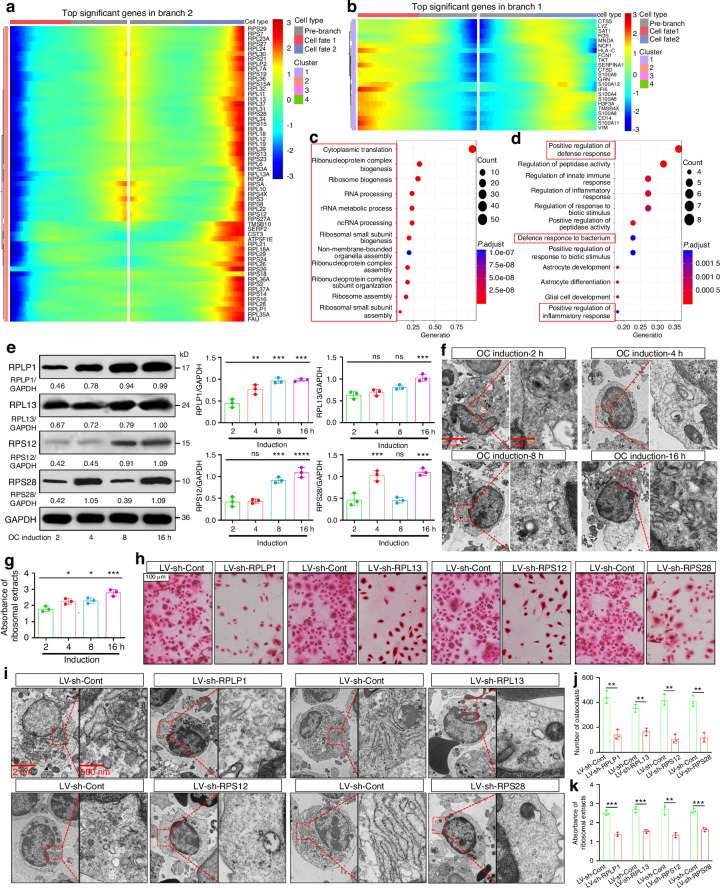
Ribosome synthesis contributes to peripheral OCP-development. **a** Heatmap showing that starting from pre-branch (middle), the top upregulated genes for fate 2 characterized by state-1 to state-3 (Namely, cell fate 1-3) were obtained compared to fate 1 (cell fate 1-2). **b** Heatmap showing that starting from pre-branch (middle), the top genes for fate 1 (cell fate 3-4) were obtained compared to fate 2 (cell fate 3-5). Red indicates the upregulated expression, while blue indicates the downregulated expression in (**a**, **b**). Functional enrichment of top genes for cell fate 1-3 (**c**) and cell fate 3-4 (**d**). The top 15 GO terms are shown. The pivotal terms are marked in red box. **e** Western-blotting analysis of RPLP1, RPL13, RPS12/28 for CD14^+^CD16^−^ monocytes at the 2^nd^, 4^th^, 8^th^ and 16^th^ hours of osteoclastic induction. **f** Representative TEM images of ribosomes for CD14^+^CD16^−^ monocytes at the 2^nd^, 4^th^, 8^th^, and 16^th^ hours of osteoclastic induction. The small black particles in the cytoplasm are ribosomes. Scale bar: 2 μm or 500 nm. Results are from three independent assays with unanimous trends. **g** Absorbance assays (260 nm) of total ribosome levels from cellular extracts. **h**, **j** Osteoclastogenic ability for RPLP1, RPL13 or RPS12/28-silenced CD14^+^CD16^−^ monocytes. Scale bar: 100 µm. **i** Representative TEM images of ribosomes for RPLP1, RPL13 or RPS12/28-silenced CD14^+^CD16^−^ monocytes at the 8^th^ hours of osteoclastic induction. The small black particles in the cytoplasm are ribosomes. Scale bar: 2 μm or 500 nm. Results are from three independent assays with unanimous trends. **k** Absorbance assays (260 nm) of total ribosome extracts from RPLP1, RPL13 or RPS12/28-silenced CD14^+^CD16^−^ monocytes at the 8^th^ hours of osteoclastic induction. *P < 0.05, **P < 0.01, ***P < 0.001, ****P < 0.000 1 and ns (not significant) by one-way ANOVA (**g**–**i**). **P < 0.01, ***P < 0.001, ****P < 0.000 1 by Student’s *t* tests (**l**, **m**)

We documented that cell-differentiation-fates 1-3 and 3-4 were involved in peripheral OCP-development. Through the analysis of differentially expressed genes (DEGs) for cell fate, we continued to explore the mysteries of peripheral OCP development. As shown in Fig. 3a, c, the upregulated genes for cell fate 1-3 participated in cytoplasmic translation, ribosome biogenesis and related functions, including *RPLP1*, *RPL13*, *RPS12*, *RPS28*, etc, which first described the responsibility of ribosome synthesis for osteoclastogenesis. Additionally, positive regulation of defense response and inflammatory response was associated with the upregulated genes for cell-fate 3-4, including *S100A9*, *S100A8*, *S100A12*, *CD14*, etc. (Fig. 3b, d). In response to various invading pathogens, host defense is involved in enhanced osteoclastogenesis in addition to pathogens themselves and secretions.^25^ Here, the involvement of host defense and relevant pro-osteoclastogenic molecules in cell development toward advanced OCPs was described. Mature osteoclasts from CD14^bright^CD16^−^ cluster were more abundant than those from CD14^dim^CD16^−^ cluster (Fig. 1d, e), consistent with reported results indicating that CD14 is beneficial for osteoclast development.^26,27^ As typical alarmins, S100A8/A9/A12 are also considered pro-osteoclastogenic factors under chronic inflammation.^28,29^ Accordingly, the significance of cell-fate 3-4 for osteoclastogenesis obtained elucidation due to its association with host defense.

Next, we explored the above inference from different perspectives. The significance of ribosome synthesis and host defense for OCP-development fate was also supported by Monocle-2-dependent dynamic analysis, revealing that according to specific gene-expression patterns, cluster-2 for state 1-3-5 lineage and cluster-1 for state 1-3-4 lineage showed overall elevation along respective pseudotime-values and the corresponding genes were involved in ribosome synthesis- and host defense-related functions, respectively (Fig. S4a–d). DEG analysis on cell-differentiation-status exhibited that the upregulated genes from state-1 to state-3 were associated with cytoplasmic translation and host defense-related functions (Fig. S4e, f); those from state-1 to overall state-3/4/5 and from state-3 to state-5 were responsible for ribosome synthesis-related functions (Fig. S4e, g, h); and cytoplasmic translation and host defense-related functions were significantly enriched in those from state-3 to state-4 (Fig. S4e, i). GSVA scores exhibited that the functions of states-3/4/5 were enhanced, and ribosome-synthesis functions of state-5 were the strongest whereas host-defense functions of state-4 were the strongest (Fig. S5a). The credibility of OCP-development fate was consolidated, further confirming the reliability of single-cell trajectory. Moreover, positive cell proportions and average expression of *RPLP1*, *RPL13* and *RPS12/28/17* were positively correlated with states-3/4/5 proportions, state-4—states-3/4/5 ratios, osteoclast proliferation, ribosome biogenesis and cytoplasmic translation; osteoclast development was positively correlated with other parameters except for *RPS12* average expression; and osteoclast differentiation was positively correlated with *RPLP1*, *RPL13* and *RPS17* expression and *RPS17-*positive cell proportions (Fig. S5b). Additionally, *CD14* and *S100A12-*positive cell proportions were positively correlated with states-3/4/5 proportions, state-4—states-3/4/5 ratios, osteoclast development/proliferation and positive regulation of defense response and inflammatory response; *CD14* expression was positively correlated with state-4—states-3/4/5 ratios, osteoclast development and positive regulation of defense response; and *CD14-*positive cell proportions were positively correlated with osteoclast differentiation (Fig. S5c). Taken together, correlation analysis revealed the role of ribosome-synthesis/host-defense functions and ribosome-synthesis-associated molecules in adjusting development direction towards OCP-lineage fate (Figs. S3a and S5b, c). Moreover, states-3/4/5, especially states-5, had higher ribosome-gene expression while *S100A8/9/12* expression in state-4 was the most obvious (Fig. S5d). Combining the correlation analysis between ribosomal molecules or host defense-related molecules and top state-genes (Fig. S5e, f), the involvement of significant molecules dominating ribosome synthesis in OCP-development is supported, and CD14 and S100A12 are additional molecules involved in OCP-development.

The involvement of ribosome synthesis in peripheral OCP development was demonstrated. The role of ribosome synthesis in OCP development needs further clarification. With osteoclastic induction, the protein levels of RPLP1, RPL13, RPS12 and RPS28 showed an overall upward trend (Fig. 3e). Furthermore, transmission electron microscope (TEM) assays showed that ribosome synthesis gradually strengthened as induction progressed (Fig. 3f). Absorbance assays from cellular extracts indicated a similar trend as the ribosome levels (Fig. 3g). These results provide strong evidence supporting our single-cell trajectory by elucidating the synchronicity between ribosome-synthesis capacity and OCP maturity. However, S100A8/9/12 protein levels lacked an overall upward trend while only CD14 protein increased in a time-dependent manner (Fig. S6a, b). Our data suggested that ribosome synthesis is an associated factor for conventional OCP development whereas the effect of host defense relies on complex in vivo environments. As shown in Figs. 3h–k and S7a, knockdown of four ribosomal molecules inhibited peripheral osteoclastogenesis and ribosome synthesis; this further clarified the contribution of ribosome synthesis to peripheral OCP development. Remarkably, knockdown of ribosomal molecules all reduced CTSK, MMP9, TRAP and NFATC1 protein expression (Fig. S7b), and NAMPT^+^ cells increased while CSTA^+^ cells decreased under RPLP1 silencing (Fig. S7c, d), supporting OCP-development fate maintained by ribosome synthesis. These results accordingly provide valuable evidence for cell-fate analysis.

### Decreased RPS17 in monocytic OCPs from AS patients

**Fig. 4:**
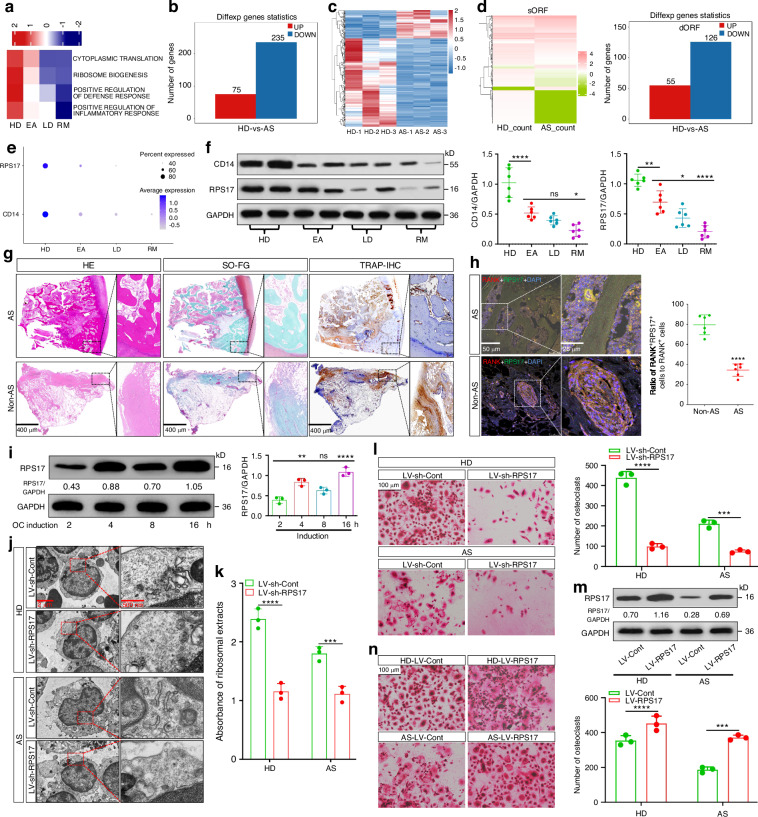
Decreased RPS17 in monocytic OCPs from AS patients. **a** GSVA scores in several functions from monocyte subsets 0/1/2/5/7/9. Rows were normalized and clustered, and the transition of blue-white-red indicates an increase in the scores. **b**, **c** Histograms (red indicates upregulation while blue indicates downregulation) and heatmap showing DEGs for different translation levels in CD14^+^CD16^−^ monocytes between AS patients and HDs revealed by Ribo-seq. **d** Heatmap (left) showing differentially expressed sORFs and histograms (right; red indicates upregulation while blue indicates downregulation) showing differentially expressed dORFs (originating from 3’-UTR region) between two groups. **e** The expression distribution of RPS17 and CD14 across four groups. Circle size indicates PCT and color depth indicates average levels. **f** Western-blotting analysis of CD14 and RPS17 for CD14^+^CD16^−^ monocytes from four groups of subjects (*n* = 6/group; each 2 samples presented in one group). **g** H&E, SO-FG and IHC (TRAP) stainings in the hip-joint tissues from AS patients and non-AS patients. Scale bar: 400 µm. **h** Representative images of RPS17-RANK coimmunostaining in the hip-joint tissues from AS patients and non-AS patients. Scale bar: 50 or 25 μm. Data in **g**, **h** come from the collected joint samples from ANFH patients (*n* = 6) and AS patients (*n* = 6) during surgeries. **i** Western-blotting analysis of RPS17 for CD14^+^CD16^−^ monocytes at the 2^nd^, 4^th^, 8^th^, and 16^th^ hours of osteoclastic induction. **j** Representative TEM images of ribosomes for RPS17-silenced CD14^+^CD16^−^ monocytes from HDs and AS patients (*n* = 3/group) at the 8^th^ hours of osteoclastic induction. The small black particles in the cytoplasm are ribosomes. Scale bar: 2 μm or 500 nm. Results are from three independent assays with unanimous trends. **k** Absorbance assays (260 nm) of ribosome extracts from RPS17-silenced CD14^+^CD16^−^ monocytes from the above subjects (*n* = 3/group) at the 8^th^ hours of osteoclastic induction. **l** Osteoclastogenic ability for RPS17-silenced CD14^+^CD16^−^ monocytes from the above subjects (*n* = 3/group). Scale bar: 100 µm. Data come from the mean values of osteoclasts in each three wells. **m** Western-blotting validation for RPS17-overexpressing CD14^+^CD16^−^ monocytes from HDs and AS patients. **n** Osteoclastogenic ability for RPS17-overexpressing CD14^+^CD16^−^ monocytes from the above subjects (*n* = 3/group). Scale bar: 100 µm. Data come from the mean values of osteoclasts in each three wells. *P < 0.05, **P < 0.01, ****P < 0.000 1 and ns (not significant) by one-way ANOVA (**f**, **i**). ***P < 0.001, ****P < 0.000 1 by Student’s *t* tests (**h**, **j**–**n**)

GSVA scores exhibited that HDs had the highest values for ribosome synthesis, and those in EAs were the highest among AS groups (Fig. 4a). These results were supported by Ribo-seq, showing that the translation levels of more genes in CD14^+^CD16^−^ monocytes from AS patients went down and compared to overexpressed small open-reading-frames (sORFs), underexpressed sORFs from AS patients, especially for downstream ORF (dORF), were more (Fig. 4b–d); this implied that weaker ribosome synthesis is responsible for AS-associated impaired OCP-development. Moreover, HDs showed the highest values in state-4—states-3/4/5 ratios and positive regulation of defense response and inflammatory response, and compared to EAs, those in LDs did not change, while those in RMs decreased (Figs. 4a and S8a), suggesting that insufficient host defense participated in impaired OCP-development under AS conditions. We documented that as key molecules for the two functions, *CD14* and *RPS17* were not only the top genes for states-4/5 but also pivotal molecules for OCP development. Notably, both *CD14* and *RPS17* were highly expressed in HDs and gradually decreased from HDs to LDs/RMs (Fig. 4e); the above trend was identified by protein detection, showing that CD14 and RPS17 expression gradually decreased from HDs to LDs/RMs (Fig. 4f). Additionally, X-rays confirmed representative hip-joint alterations for AS, such as joint erosion and fusion, joint-space narrowing and femoral head destruction, and histology assessment showed that AS-joint tissues presented massive new-bone formation adjacent to fused joint besides disappeared joint spaces (Figs. 4g and S8b). Although TRAP was expressed to a certain extent in the new bones located in AS-joint tissues (Fig. 4g), RPS17 fluorescence expression in bone-surface-OCPs (displayed as RPS17-RANK overlapping fluorescence) was significantly weaker than that in subchondral bones of non-AS patients with ANFH (Figs. 4h and S8c), inferring that abnormal RPS17 expression exists in AS-joints, resulting in insufficient abundance of osteoclasts to absorb new bones. The above results reveal that impaired osteoclastogenesis associated with AS is attributed to low CD14 and RPS17 expression, and insufficient RPS17 participates in aberrant osteogenesis and pathological changes for AS patients. Immunofluorescence staining also demonstrated the preferred distribution of RPS17 in OCP-featured states (Fig. S8d). With osteoclastic induction, RPS17 protein expression in CD14^+^CD16^−^ monocytes showed an overall increasing trend (Fig. 4i); this revealed that as an essential ribosomal molecule, RPS17 participates in conventional OCP-development. As expected, RPS17 silencing reduced ribosome synthesis and peripheral osteoclast abundance for HDs and AS patients (Figs. 4j–l and S8e). RPS17 overexpression not only enhanced osteoclastogenic ability for HDs but also recovered the reduction in that of AS patients, thereby confirming RPS17-promoted osteoclastogenesis (Fig. 4m, n). Additionally, RPS17 did not interact with other key osteoclast-regulating factors upon osteoclastic induction, including RANK, NFATc1, c-Fos and DCSTAMP (Fig. S8f); this suggested the specificity of OCP-development fate maintained by ribosome synthesis for RPS17-promoted osteoclastogenesis mechanistically.

### RPS17-CKO ameliorates osteoclastogenic bone loss

**Fig. 5:**
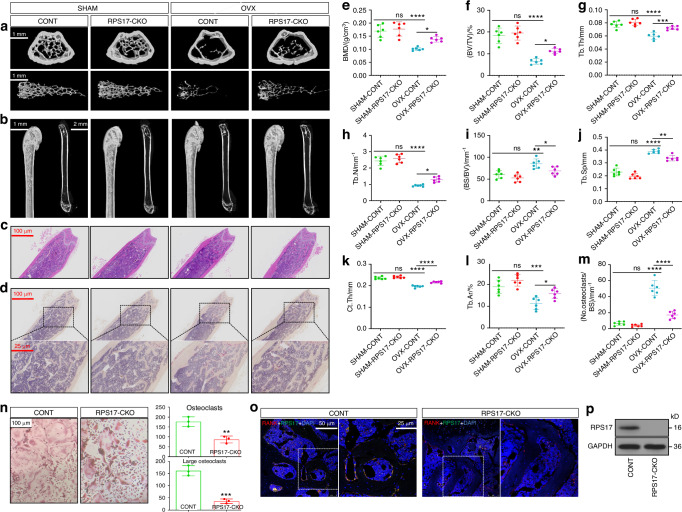
RPS17-CKO ameliorates osteoclastogenic bone loss in OVX mice. **a**, **b** Representative 3D Micro-CT reconstructed images of femurs from 16-weeks old RPS17-CKO mice and control mice treated with sham surgery or ovariectomy showing bone mass and bone microstructure (*n* = 6/group). Scale bar, 1 or 2 mm. **c** Representative H&E-stained tibial sections from each group. Scale bar, 100 μm. **d** Representative TRAP-stained tibial sections from each group. Scale bar, 100 or 25 μm. **e**–**k** The trabecular bone parameters (BMD, BV/TV, Tb.Th, Tb.N, BS/BV and Tb.Sp) and cortical bone parameter (Ct.Th) were analyzed using Micro-CT scanning. **l** Tb.Ar was analyzed via H&E staining and IPP system. **m** The number of osteoclasts per millimeter (mm) for trabecular bone surface was counted. Experiments were repeated in each group of mice independently. **n** Osteoclastogenic ability of OCPs from RPS17-CKO mice and control mice, including the formation of mature osteoclasts and large osteoclasts. Scale bar: 100 µm. **o** Representative images of RPS17-RANK coimmunostaining from RPS17-CKO mice and control mice. Scale bar: 50 or 25 μm. **p** Western-blotting analysis of RPS17 in OCPs from RPS17-CKO mice and control mice. *P < 0.05, **P < 0.01, ***P < 0.001, ****P < 0.000 1 and ns (not significant) by one-way ANOVA (**e**–**m**). **P < 0.01; ***P < 0.001 by Student’s *t* tests (**n**). SHAM sham surgery, OVX ovariectomy, CONT control mice

The role of RPS17 in osteoclastogenesis was documented. We attempted to determine the significance of RPS17 in osteoclastogenic bone loss through the investigation of RPS17-conditional knockout (RPS17-CKO: RPS17^flox/flox^; LysM-Cre) mice. Through phenotype observation, we found that although RPS17-CKO did not affect the physiological phenotype of mice, it improved bone damage in ovariectomized (OVX) mice. Micro-CT scanning displayed that compared to control mice in the same litter (RPS17^flox/flox^), bone loss and trabecular bone damage in OVX mice with RPS17-CKO were markedly ameliorated (Fig. 5a, b); this was also reflected in increased BMD, BV/TV, Tb.Th and Tb.N as well as decreased BS/BV and Tb.Sp upon RPS17-CKO (Fig. 5e–j). Moreover, decreased Ct.Th in OVX mice also increased with RPS17-CKO (Fig. 5k). Additionally, H&E staining displayed that the reduction in the trabecular area (Tb.Ar) of OVX mice was partially blocked with RPS17-CKO (Fig. 5c, l). Importantly, TRAP staining showed that the sharply increased abundance of osteoclasts in the trabecular bones of OVX mice decreased significantly with RPS17-CKO (Fig. 5d, m). Although there was no significant difference in the above parameters between sham-operated (SHAM) mice with RPS17-CKO and control mice, improved bone loss and inhibited osteoclastogenesis by RPS17-CKO in vivo confirmed the significance of RPS17 in osteoclastogenesis. Furthermore, osteoclastogenic capacity of RPS17-knockout OCPs also sharply decreased upon induction (including mature osteoclast number and size) (Fig. 5n), consistent with in vivo results, further confirming the contribution of RPS17 to osteoclastogenesis. The knockout efficiency of RPS17 was confirmed by immunofluorescence staining and in vitro protein detection (Figs. 5o, p and S9); this validated the reliability of the in vivo assays. Accordingly, a novel anti-osteoclastogenic/osteoporotic target, RPS17, was discovered.

### RPS17 overexpression alleviates the phenotype for AS modeling mice

**Fig. 6:**
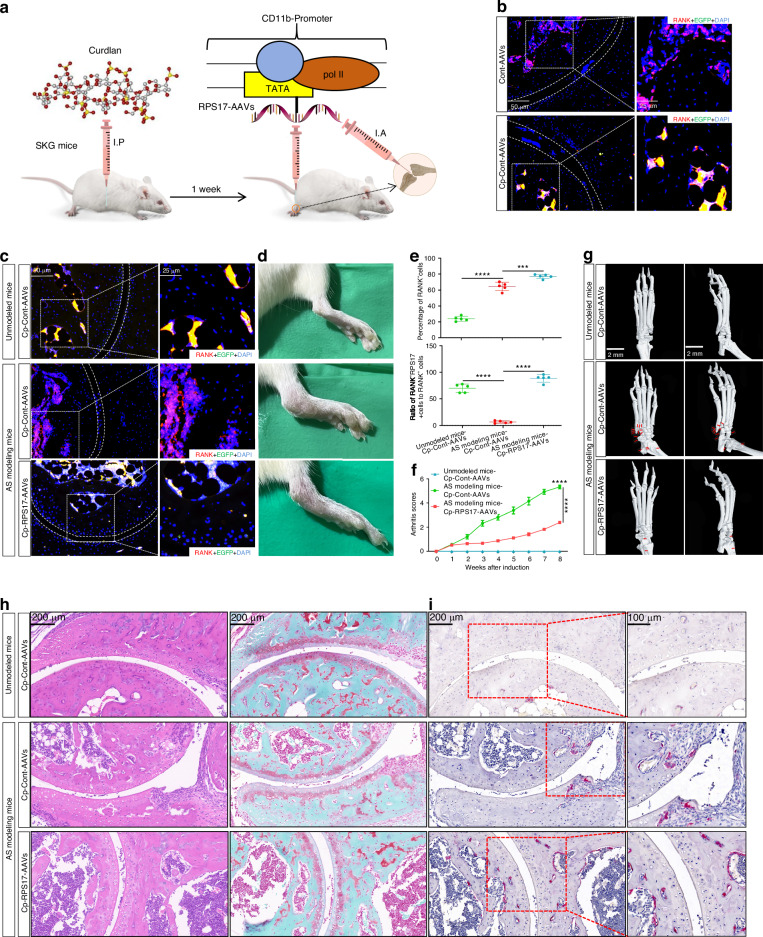
RPS17 overexpression alleviates the phenotype for AS modeling mice. **a** The administration model of CD11b-promoter-RPS17-AAVs in AS modeling mice (using SKG mice). After 1 week of curdlan induction, the above AAVs were used to treat SKG modeling mice by local injection into ankle-joint cavity. **b** Representative images of EGFP-RANK coimmunostaining in the ankles of SKG mice treated with EGFP-labeled conventional AAVs or CD11b-promoter-loaded AAVs. Scale bar: 50 or 25 µm. **c** Representative images of RANK-RPS17 coimmunostaining in the ankles of SKG modeling mice treated with Cont-AAVs or RPS17-AAVs (*n* = 5/group). Scale bar: 50 or 25 µm. Unmodeled mice were used as normal controls. **d** Comparison of ankle-swelling degree between three groups of mice (*n* = 5/group). **e** Quantitative comparison of RANK or RPS17 fluorescence expression in subchondral bones between groups (*n* = 5/group). **f** Mice from each group were evaluated with clinical scores once a week, and the overall trend was compared between groups (*n* = 5/group). **g** Micro-CT showing the formation of osteophytes (red arrows) in the ankle joints and joint-degeneration degree across each group (*n* = 5/group). Scale bar: 2 mm. **h** H&E or SO-FG staining showing ankle-joint destruction and ossification across each group (*n* = 5/group). Scale bar: 200 μm. **i** TRAP staining showing osteoclastogenic levels for new bones adjacent to articular surface (*n* = 5/group). Scale bar: 200 or 100 μm. ***P < 0.001, ****P < 0.000 1 by one-way ANOVA. Cp-Cont-AAVs CD11b-promoter-Cont-AAVs, Cp-RPS17-AAVs CD11b-promoter-RPS17-AAVs, I.P intraperitoneal injection, I.A intra-articular injection

The contribution of RPS17 to osteoclastogenesis was identified. To confirm the role of RPS17 in AS lesions, we applied RPS17-cDNA-Adeno-associated virus 9 with CD11b-promoter (CD11b-promoter-RPS17-AAVs), which can specifically overexpresses RPS17 in circulating OCPs,^30,31^ to treat AS modeling mice by intra-articular injection in ankle joints (Fig. 6a). Immunofluorescence staining displayed that CD11b-promoter-loaded AAVs could more effectively carry AAVs (coupling EGFP) into RANK^+^ area (Figs. 6b and S10a); this confirms the effectiveness of CD11b-promoter-AAVs in targeted delivery into OCPs. Compared to unmodeled mice, AS modeling mice showed strong RANK fluorescence in the entire subchondral bones but weak RPS17 fluorescence (represented as dual fluorescence of RPS17 and RANK) (Figs.6c, e and S10b). However, both types of fluorescence were strengthened with RPS17-AAV injection (Figs. 6c, e and S10b). Therefore, RPS17 expression alterations in AS patients were validated at the animal level, which not only supports the key findings of this study but also confirms the availability of AS modeling mice. Furthermore, the effectiveness of RPS17-specific overexpression was identified. Ankle-joint swelling degree in AS modeling mice was markedly improved by RPS17-AAVs (Fig. 6d). The arthritis scores also decreased after treatment (Fig. 6f). Micro-CT results showed that RPS17-AAVs effectively reduced joint-degeneration degree and improved osteophyte formation in the ankles (Fig. 6g). Histology assessment showed that RPS17-AAVs alleviated ankle-joint-destruction degree (Fig. 6h). Notably, different from unmodeled mice, AS modeling mice showed bulk new bones adjacent to articular surface, with a certain amount of osteoclasts (Fig. 6h, i). Nevertheless, both decreased new bones and increased osteoclasts were observed in RPS17-overexpressed mice (Fig. 6h, i). The relationship between RPS17-mediated osteoclastogenesis and AS-like lesions was supported.

### The therapeutic value of RPS17-overexpressed monocytic OCPs in AS-like lesions

**Fig. 7:**
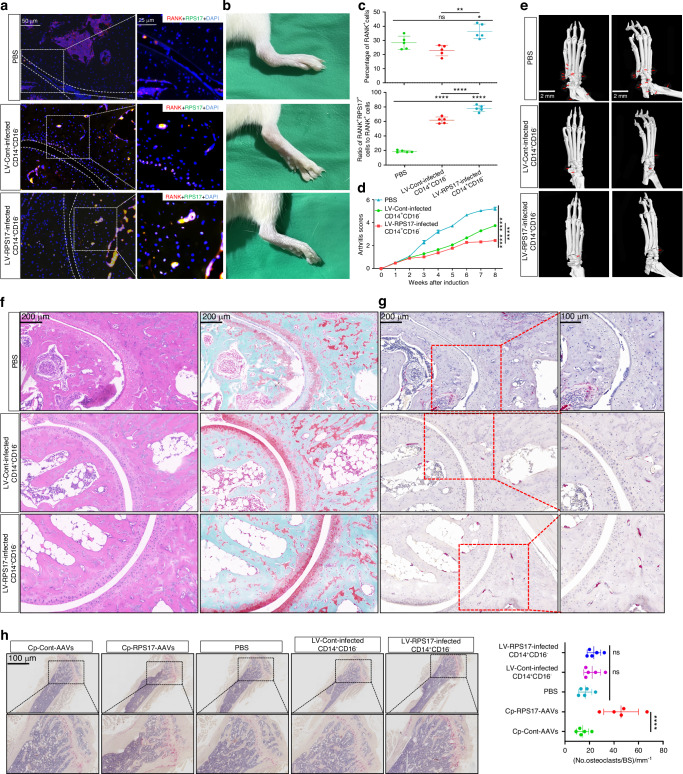
The therapeutic value of RPS17-overexpressed monocytic OCPs in AS-like lesions. **a** Representative images of RANK-RPS17 coimmunostaining in the ankles of SKG modeling mice injected with PBS, control CD14^+^CD16^−^ monocytes or RPS17-overexpressed cells in ankle-joint cavity (*n* = 5/group). Scale bar: 50 or 25 µm. **b** Comparison of ankle-swelling degree between groups (*n* = 5/group). **c** Quantitative comparison of RANK or RPS17 fluorescence expression in bones adjacent to articular surface between groups (*n* = 5/group). **d** Mice from each group were evaluated with clinical scores once a week, and the overall trend was compared between groups (*n* = 5/group). **e** Micro-CT showing the formation of osteophytes (red arrows) in the ankle joints and joint-degeneration degree across each group (*n* = 5/group). Scale bar: 2 mm. **f** H&E or SO-FG staining showing ankle-joint destruction and ossification across each group (*n* = 5/group). Scale bar: 200 μm. **g** TRAP staining showing osteoclastogenic levels for new bones adjacent to articular surface (*n* = 5/group). Scale bar: 200 or 100 μm. **h** TRAP staining showed osteoclastogenic levels in the trabecular bones across each group, and quantitative evaluation relied on counting the number of osteoclasts per mm of bone surface (BS) (*n* = 5/group). Scale bar: 100 or 25 μm. *P < 0.05, **P < 0.01, ***P < 0.001, ****P < 0.000 1 and ns (not significant) by one-way ANOVA

Given the risk of RPS17 overexpression in exacerbating bone loss, we should seek a strategy that is both effective and safe. Due to the difficulty of cells being absorbed and entering subchondral bones, cytotherapy has strong safety and translational value. Furthermore, bone-marrow OCPs, due to their similarity to monocytic myeloid-derived suppressor cells (M-MDSCs), exert resistance against inflammatory arthritis through T-cell-suppressive activity.^32^ We discovered that RPS17-overexpressed CD14^+^CD16^−^ monocytes exhibited stronger expression of M-MDSC-markers (CD11b, CD33 and SPARC) that are reported previously,^33,34^ and significantly inhibited T-cell proliferation (Fig. S11). The potential of monocytic OCPs, especially RPS17-overexpressed cells, to suppress joint immune inflammation was suggested. Compared to PBS-treated or control cells-treated mice, the mice administered by RPS17-overexpressed cells showed stronger RANK and RPS17 fluorescence in bone tissues adjacent to articular surface (Figs. 7a, c and S12); this validated the usability of monocytic OCPs. As expected, subchondral bones in cells-treated mice did not exhibit large-scale RPS17 fluorescence (Figs. 7a and S12); this demonstrates one of the advantages of cytotherapy that it tends to exert the effects around the joints. RPS17-overexpressed cells also had stronger abilities than control cells in alleviating ankle-joint swelling and decreasing arthritis scores (Fig. 7b, d). Micro-CT displayed that both cytotherapies effectively improved joint degeneration and osteophyte formation in the ankles (Fig. 7e). Histology assessment displayed that both cytotherapies avoided marked joint destruction (Fig. 7f). Notably, different from control mice, cells-treated mice lacked bulk new bones adjacent to articular surface (Fig. 7f, g). RPS17-overexpressed cells generated more osteoclasts in the adjacent bones than those of control cells (Fig. 7g). Nevertheless, there was no obvious increase in osteoclasts in subchondral bones with cell intervention (Fig. 7g), indicating that cytotherapy can evade excessive osteoclastic activation in subchondral bones. Notably, Micro-CT displayed that RPS17-AAVs reduced bone mass in AS modeling mice while both cytotherapies had no significant effects (Fig. S13a); this was also reflected in decreased BMD, BV/TV, Tb.Th and Tb.N as well as increased BS/BV and Tb.Sp after RPS17-AAV administration (Fig. S13b). Importantly, the trabecular bones in cytotherapy groups did not exhibit increased osteoclasts that was observed under RPS17-AAV treatment (Fig. 7h). Therefore, cytotherapy, rather than RPS17-specific overexpression, can avoid bone loss. Additionally, no obvious graft-versus-host disease (GVHD) was found during all experiments, such as rash, diarrhea, anorexia, etc. Moreover, the body weight and several blood indicators of mice showed non-significant alterations with cell transfer (Fig. S13c). Therefore, the interference of heterologous immune rejection was excluded. These data demonstrate that cytotherapy can improve the joint phenotype of AS-like mice while maintaining the safety. RPS17-overexpressed OCPs exhibit stronger capacity than control cells in resisting inflammation and promoting local osteoclastogenesis; this highlights the upgraded effect of genetic modification on cytotherapy.

## Discussion

As a typical rheumatic disease, AS is typically characterized by local new-bone formation, which causes ankylosing malformation.^35^ New-bone excessive formation requires the support of weak osteoclastogenesis. In addition to bone-resorbing cell characteristic, microRNAs from osteoclast-derived extracellular vesicles were also demonstrated to inhibit osteoblast differentiation in vitro and bone formation in vivo.^36^ However, current AS research focuses on aberrant osteogenesis that relies on osteoblasts,^7–9^ with limited studies concerning the effect of osteoclasts.^17^ Addressing human in vivo peripheral OCP-development patterns is conducive to investigating osteoclast-associated AS inflammatory osteogenesis. Previous literatures discussed the osteoclastogenic potential of peripheral OCPs, phenotypic markers, possible sources, etc.^18,19,23,24,30,37^ However, the evolutionary pattern of peripheral OCPs towards mature osteoclasts is still unclear. For example, why do some monocytes differentiate into OCPs while other cells follow the conventional pathway? Which population of monocytes is closer to OCPs? CD14^+^CD16^−^ monocytes are recognized as the main human peripheral OCPs.^18,19,23,24^ Therefore, based on the biological information contained in CD16^dim^ monocytes in our scRNA-seq, peripheral OCP-development patterns can be well elucidated.

Firstly, different from the directional differentiation of C monos to osteoclasts, the strong osteoclastogenic potential of 14^dim^16^dim^ monocytes is attributed to their location in the early stage of differentiation; this leads them to have an advantage over other clusters in terms of fusion and differentiation towards multinucleated osteoclasts. Subsequently, the overall trajectory of peripheral OCP-development was unmasked *via* the clarification of pseudotimeline. Here, single-cell trajectory of monocytic OCPs is the first to reveal the cell states more inclined towards OCP-lineage fate, by which OCPs present definite differentiation fates starting from corresponding nodes. On this basis, the two novel functions that govern OCP-lineage-fate commitment, ribosome synthesis and host defense, entered our vision; this discovery was consistently supported by three trajectory-based analyses. We were accordingly reminded that ribosome synthesis- and host defense-related functions collectively promote OCP-development by directing cell fate, advancing differentiation trajectories and transforming cell states. Remarkably, among two OCP-featured states, one is mainly dominated by ribosome synthesis while the other receives the combined influence of both functions. Therefore, ribosome synthesis is an essential factor for OCP-development, while host defense is an additive factor for the production of more efficient OCPs. Ribosome synthesis drives cell growth. The maintenance of cell pluripotency, commitment to a specific cell fate and transition towards cell differentiation depend on ribosome synthesis accompanied by protein translation;^38,39^ these theories conform to our trajectory analysis for cell fate and multiple experimental evidence. Together, our data demonstrated that ribosome synthesis lays the foundation for peripheral OCP-maturation based on specific monocyte fate; this was also supported by strong correlation between multiple ribosomal molecules and OCP-development parameters from different individuals. In vitro assays suggested that unlike ribosome-synthesis levels synchronized with OCP-development under conventional induction, host defense tends to be driven by in vivo inflammatory environments; this elucidates the difference between the two functions. Multiple molecules dominating the two functions are accordingly potential therapeutic targets for abnormal bone resorption. Our discovery implemented an integral description for the landscape of peripheral OCP-development; this sheds light on mysterious osteoclastogenic behavior, thereby providing important references for addressing osteoclast-induced bone destruction.

**Fig. 8:**
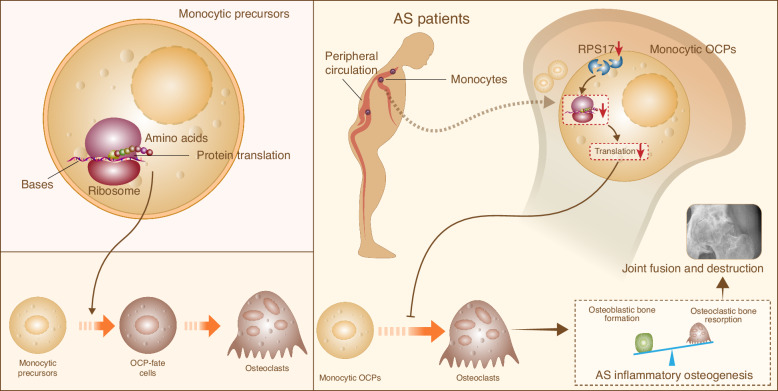
Schematic diagram showing the significance of RPS17-ribosome synthesis-osteoclastogenesis signal transmission for inflammatory osteogenesis and joint damage upon AS conditions. Ribosome synthesis in monocytic precursors promotes the development of monocytic precursors towards OCP-fate cells and subsequent osteoclast differentiation by driving protein translation. Upon AS conditions, decreased RPS17 in monocytic OCPs from the periphery leads to a decline in ribosome synthesis and translation function, which inhibits monocytic osteoclastogenesis; this causes an imbalance of bone turnover, i.e. excessive bone formation, thereby resulting in aberrant osteogenesis as well as fusion and destruction of the involved joints

Importantly, the alterations of peripheral OCP-development across AS conditions obtained elaboration, suggesting that fewer and fewer cells developed toward OCP-lineage fate in the initial and deterioration/remission stages, conforming to previous study describing that monocytic osteoclastogenesis for AS patients is weaker and osteoclast abundance decreases year by year as disease continues.^20^ The two functions and their representative molecules (CD14 and RPS17) showed a significant positive correlation with osteoclastogenic capacity, and decreased as impaired osteoclastogenesis under AS onset and different outcomes; this discovery provides important insights into AS aberrant osteogenesis. We believe that underexpressed CD14 or RPS17 in AS patients inhibits osteoclastogenesis by suppressing respective functions, thereby promoting inflammatory osteogenesis. Especially, the involvement of RPS17 underexpression in AS inflammatory osteogenesis and ankylosing destruction was supported in human and animal histology. As a key molecule mediating ribosome synthesis, the role of RPS17 in osteoclastogenesis and OVX-induced bone loss was confirmed; this presents important target for the treatment of osteoclastic bone loss. As known, RPS17 functions in ribosome synthesis through the assembly of ribosomal small subunits.^40^ Therefore, RPS17-dominated ribosome synthesis may contribute to OCP-lineage commitment and osteoclastogenesis through a series of protein expression downstream. Nevertheless, the above process is compromised under AS conditions; this causes massive new-bone formation through attenuated bone resorption at lesion sites. The role of ribosome in osteoclast differentiation and the effects of underexpressed RPS17 on monocytic osteoclastogenesis and subsequent abnormal osteogenesis upon AS conditions were described in Fig. 8. The role of RPS17 in improving AS lesions was supported by SKG modeling mice with RPS17 overexpression, which was indeed based on enhanced osteoclastogenesis. The translational potential of RPS17 was accordingly highlighted. The reliance of RPS17-specific overexpression on CD11b-promoter is due to CD11b being a marker for mouse circulating OCPs;^30,31^ this brings animal experiments closer to the main theme of our study. Nevertheless, both new-bone formation and systemic bone loss occur in AS.^35^ RPS17 overexpression targeting bone-resorption area is not an appropriate strategy for AS treatment due to the risk of exacerbating bone loss. Based on RPS17-overexpressed monocytic OCPs, we designed a safe and effective cytotherapy regimen that effectively improves arthritis, joint destruction and ossification while avoiding further reduction in bone mass and obvious side effects. To escape from the risk of extensive osteoclastic activation, local injection into joint cavity is used as administration route. Adopting intra-articular channel is prompted by previous research describing that monocyte-derived macrophages on inflammatory synovium have strong osteoclastogenic capacity,^41,42^ suggesting that intra-articular injection is beneficial for the targeted contact between monocytic OCPs and AS new-bones; its feasibility has also been validated by empirical evidence. Upon AS conditions, the developmental pathway from monocytes to osteoclasts through intra-articular injection deserves future exploration. Nevertheless, the above regimen provides meaningful references for the treatment of AS inflammatory osteogenesis from osteoclastogenic perspective. The reinfusion of monocytic OCPs from patients into the affected joints after genetic modification may become an option for improving AS peripheral lesions in future; this endows our cytotherapy with therapeutic prospect. Remarkably, the anti-arthritis function of monocytic OCPs, especially RPS17-overexpressed cells, is associated with their T-cell-suppressive property; this is inspired by previous literature showing OCPs carrying M-MDSC attribute.^32^ This study also attempted to transfer bone-marrow OCPs into an absorptive arthritis model similar to rheumatoid arthritis (RA), and found that OCPs improved arthritis without exacerbating absorption.^32^ But due to the synchronous requirement for bone-resorption prevention and anti-inflammatory environment, designing a strategy to prevent OCP from developing into osteoclasts is more necessary for RA-joint treatments. However, monocytic OCPs that combine pro-osteoclastogenic and anti-inflammatory activities are more likely to directly function in AS-joints characterized by inflammatory osteogenesis. Here, we propose a novel cytotherapy based on enhancing new-bone resorption. To advance its translational research and clinical applications, many issues remain to be clarified. To avoid damaging normal bones and promote the clinical application, corresponding treatment strategies should be improved in future, thereby forming standardized guidelines. The application of biological materials, including nanoparticles, also deserves further research. On this basis, the comparison between this cytotherapy with current AS treatments such as TNF or IL-17 inhibitors, as well as exploring combination therapies, is indispensable and valuable. However, the long-term safety evaluation of this cytotherapy should be taken seriously. Notably, an increase in the frequency of bone-density testing can help monitor the risk of bone loss upon therapy. Moreover, the sample size of healthy donors is relatively small, and future studies with larger cohorts are needed to validate the generalizability of the findings.

Notably, total OCPs consist of bone-marrow OCPs and circulating OCPs, both of which are responsible for bone resorption. Bone-marrow OCPs directly localize in osteoclast niches within bone-marrow cavity to contribute to systemic bone turnover, while circulating OCPs are more likely to migrate to specific locations through bone vessels. AS is characterized by strong peripheral inflammation and local pathological alterations. In this context, peripheral OCP-changes are more inclined to participate in AS lesions and their calibration is more in line with the need for improving conditions; this hypothesis is supported by our data. Meanwhile, bone-marrow OCPs continue to participate in inflammatory bone loss. Accordingly, we infer that both AS deterioration and remission present continuously weakened osteoclastogenesis, either of which induces ankylosis and combats bone loss, respectively; this reminds us that different strategies should be adopted according to different conditions. Based on immunocyte scRNA-seq, our experimental evidence not only provides a multi-dimensional analysis for osteoclastogenesis rarely studied in AS, but also makes a detailed clarification to previous preliminary findings.^20^

Based on multicellular descriptions, we presented a comprehensive view for monocyte-related immunological profiles across AS conditions. Importantly, the comprehensive analysis based on single-cell trajectory revealed peripheral OCP-development patterns describing that ribosome synthesis is the essential factor for the development from monocytic precursors towards OCPs and host defense promotes advanced OCP-development. The reduction in OCP development upon AS conditions was further described; this is attributed to decreased dominant molecules for the two functions. Importantly, the significance of RPS17-dependent ribosome synthesis was emphasized. Furthermore, the therapeutic value of monocytic OCPs was uncovered. We believe that intra-articular injection of RPS17-overexpressed monocytic OCPs has the potential to prevent AS peripheral lesions. Additionally, a decrease in CD14-dependent host defense should also contribute to damaged osteoclastogenesis in AS patients; this requires the support of detailed functional study in future. Overall, our research links monocytic immunity with AS-associated bone turnover, thereby serving as an important reference for understanding human osteoclastogenic behavior and AS aberrant osteogenesis in depth and contributing to the improvement in treating osteoclastic bone destruction and AS.

## Materials and methods

### Patient recruitment

Three HLA-B27-positive HDs with matching age were enrolled via genetic screening, and fourteen AS patients were recruited at Fujian Provincial Hospital from May to July 2021. All AS patients satisfied the modified New York (NY) criteria, and the categorization of three disease groups (EA, LD and RM) was according to clinical hallmarks and imaging evidences. The five patients in the EA group were in the early stage of disease, showing obvious symptoms, and had not yet received standard treatments, including various NSAIDs and DMARDs. The five patients in the LD group were all disabled, and confirmed by radiographic evidence of structural damage in hip joints and spinal deformity. The four patients in the RM group received standardized treatment under the guidance of specialist doctors, and maintained clinical remission and normal laboratory indicators for over 6 months (simultaneously meeting BASDAI ≤ 3, CRP ≤ 3.5 mg/L and ESR ≤ 15 mm/h). Patients in both EA and RM groups were confirmed to have no structural damage or deformities by radiographic evidences. Additionally, twelve surgical patients (6 cases of AS with damaged/fused hip joints and 6 cases of non-AS with avascular necrosis of femoral head (ANFH)) were enrolled from 2021 to 2023. With ethics committee approval (K2022-09-055) and patient consent, peripheral blood was collected from three groups of patients and three HDs mentioned above for single-cell RNA sequencing (scRNA-seq), and the femoral head samples were collected during surgeries. The six additional subjects per group (including HLA-B27-negative HDs) underwent blood testing from 2021 to 2024. The clinical features of all subjects are presented in Tables S1–3.

### scRNA-seq of PBMCs

The blood samples in the dedicated separation tubes (BD Vacutainer® CPT™ Cell Preparation Tube, 362761, BD Biosciences, NJ, USA) were centrifuged at 1 500 × *g* for 30 min at 20 °C to obtain PBMCs. Cells from each sample showed a viability rate over 90% and an aggregation rate under 5%. Using single-cell 3 ‘Library and Gel Bead Kit V3.1 and Chromium Single Cell G Chip Kit (10x Genomics; 1000121, 1000120), cell suspension was loaded onto the Chromium single cell controller to generate single-cell gel beads in the emulsion according to manufacturer’s protocols. The captured cells were lysed and the released RNA was barcoded through reverse transcription in individual GEMs. According to manufacturer’s introduction, scRNA-seq libraries were constructed using Single Cell 3’ Library and Gel Bead Kit V3.1. scRNA-seq data were normalized with LogNormalize (scale factor 10 000). Two thousand highly variable genes were identified, and normalized counts were scaled using ScaleData. Batch effects among all samples were alleviated with Harmony. Cellranger-6.0.1 software was obtained from 10x Genomics website (https://support.10xgenomics.com/single-cell-gene-expression/software/downloads/latest.) Seurat R package (v.4.0.0) was used to assemble multiple distinct scRNA-seq datasets into an integrated and unbatched dataset. After nonlinear dimensional reduction and projection of all cells into two-dimensional space through UMAP, cells are clustered together based on common features. We used built-in “FindMarkers” functions with Wilcoxon Rank Sum test in Seurat to identify DEGs in scRNA-seq analyses. The thresholds for DEG screening are: percentage of expressed cells (min.pct>0.1), Log_2_ fold change (log_2_FC ≥ 0.5), Adjusted *P* value (*P*_adj≤0.05). “FindAllMarkers” function in Seurat was employed to find the markers for each identified cluster. The Metascape network tool (www.metascape.org) was used to analyze the functional enrichments of top genes. Datasets originated from GO-Biological Process.

### Reclustering of monocytes

Five adjacent clusters based on UMAP that conform to the gene expression distribution of monocytes were extracted and integrated for reclustering. After integration, genes were scaled to unit variance. Principal component analysis and clustering were performed as described above. Then, clusters were merged and annotated according to the expressions of typical markers for specific cell types.

### GSVA functional scorings

GSVA was used to evaluate functional enrichment scores between each group based on the gene expression in the gene-sets. When analyzing individual gene-sets, the preprocessed log_2_ gene expression matrix for each gene-set was used as the GSVA input. GSVA runs separately on each gene-set. Each signature requires a minimum of 2 genes.

### Single-cell trajectories analysis

Single-cell trajectories were built with Monocle-2 (R package) that introduced pseudotime, which was used to analysis the differentiation fate of peripheral OCPs (mono subsets 0, 1, 2, 5, 7, 9). Genes were filtered by the following criteria: expressed in more than 10 cells; average expression value greater than 0.1; *Q* value less than 0.01 in different analysis. After obtaining pseudotime results, further analysis related to differentiation nodes were performed to investigate key genes determining differentiation directions. Branched expression analysis modeling was used to analyze cell data and indicated nodes following pseudotime sorting, thereby discovering DEGs related to branching. Then, the top100 DEGs were selected for functional enrichments.

When performing pseudotime-DEG analysis on state 1-3-4 lineage and state 1-3-5 lineage, the required cells were extracted from the CellDataSet based on their states. Then, pseudotime-DEG analysis was performed using the differentialGeneTest function, with only order genes considered during testing. After completing the differential analysis, the genes with qval less than 0.05 and expressed in at least 50 cells were selected for visualization using visCluster from ClusterGVis function (https://github.com/junjunlab/ClusterGVis) package and the top 10 genes of each gene cluster were labeled. Finally, based on the visualization results and qval, the top 100 genes that were overall upregulated along pseudotime values were selected and subjected to functional enrichments using ClusterProfiler (cluster-2 for state 1-3-5 lineage and cluster-1 for state 1-3-4 lineage).

When conducting DEG analysis on cell differentiation status for state-3 vs. state-1, state-4 vs. state-3, state-5 vs. state-3, overall state-3/4/5 vs. state-1, the subsets of CDS were taken based on order gene and state comparison, and differential analysis was completed by differentialGeneTest (fullModelFormulaStr = “~Group”). When calculating the average expression value of each gene in each state, the expression values were normalized using sizeFactors, and then the average value was calculated based on the grouping by state. Subsequently, genes with *q*val<0.05 were selected, and the top 100 genes were selected for enrichment analysis based on the difference in average expression between the experimental and control groups. Additionally, we also selected the top 10 genes and visualized them using the plot_genes_jitter function.

### Ribosome profiling sequencing (Ribo-seq)

After cell fixation, collection and lysis, the recovered supernatant was treated with 10 μL RNase I and 6 μL DNase I, and subjected to ribosomal isolation in MicroSpin S-400 Column and ribosomal protective fragment (RPF) extraction in spin column. The remains were subjected to PCR reaction at 68 °C for 10 min to remove rRNA (4 μL Ribo-Zero rRNA Reaction Buffer + 8 μL Ribo-Zero Removal Solution + 28 μL RNA sample). Finally, the library was constructed using High Sensitivity DNA assay Kit (Agilent Technologies, Inc.). The input data for intergroup differential expression analysis is the reads count data obtained from gene expression analysis using DESeq2 software. Based on the results of differential analysis, we selected genes with FDR < 0.05 and |log_2_FC | >1 as significant DEGs. Next, the translation abundance of sORFs encoding short peptides identified was calculated and quantified. According to the abundances of ribosome footprints (RFs) in various sORFs, the translation-difference-multiples of sORFs between groups were statistically analyzed, and DESeq2 was used to analyze the significance of expression differences in sORFs. Finally, we used |log_2_FC | ≥1.5 and *P* < 0.05 as the criteria for differential sORFs, and further calculated the translation-difference-multiples of downstream ORF (dORF: originating from 3 ‘ UTR region).

### Cell enrichments through bead sorting and FACS

For MicroBead sorting, PBMCs were incubated with CD3 (130-050-101, Miltenyi), CD19 (130-050-301, Miltenyi) MicroBeads and NKp46 (CD335)-coupled anti-Biotin MicroBeads (130-125-207/130-090-485, Miltenyi), which ran over LS column (130-042-401, Miltenyi) in deplete mode. Then, after blockage of nonspecific binding using Fc Receptor Blocking Solution (Human TruStain FcX™, BioLegend, San Diego, CA, USA), cells were resuspended in Dulbecco’s Phosphate-Buffered Sallines (DPBS) supplemented with 10% bovine serum albumin (BSA) and stained for CD14-APC (301808, Biolegend), CD16-FITC (360716, Biolegend) for 20 min on ice. CD14^+^CD3^−^CD19^−^NKp46^−^ cells were regarded as monocytes.^22^ And individual cell populations were sorted according to CD14 and CD16 expressions within CD14^+^ gate using BD FACSAria III (BD Biosciences). Additionally, cells were stained for CD3-FITC (317306, Biolegend), CD4-APC (300514, Biolegend) and CD8a-APC (300912, Biolegend) antibodies to enrich CD4^+^ T cells and CD8^+^ T cells.

### Lentiviral infection

Lentiviral vectors encoding human RPLP1-shRNA, RPL13-shRNA, RPS12-shRNA, RPS28-shRNA and RPS17-shRNA/cDNA were purchased commercially (cDNA: pRRLSIN-cPPT-SFFV-MCS-3FLAG-E2A-EGFP-SV40-puro; shRNA: pRRLSIN-cPPT-U6-shRNA-SFFV-EGFP-SV40-puro; Genechem, Shanghai, China). CD14^+^CD16^−^ monocytes under fine condition were seeded onto 6-well plates and incubated at 37 °C in 5% CO_2_ overnight. Cells (1.2 × 10^4^ cells/well) were infected for 24 h within the viruses in the presence of polybrene. Then, original medium was replaced by the medium containing fresh FBS. Three days later, antibiotic selection was conducted by adding puromycin into medium during each replacement, lasting for 7 days prior to expansion. The transduction efficiency was validated by Western blotting. According to Western-blot results, the silencing efficiency of LV-sh-RPLP1-3, LV-sh-RPL13-1, LV-sh-RPS12-3, LV-sh-RPS28-3 or LV-sh-RPS17-3 was considered the strongest. Accordingly, cells transduced by the optimum shRNAs were incubated within induction medium to induce mature osteoclasts.

### Osteoclastic differentiation assay

Various enriched monocytes (96-well plates, 10 000 cells/well) were cultured in complete α-Minimum Essential Medium (MEM) (containing 10% FBS, penicillin, streptomycin and L-glutamine) with 30 ng/mL M-CSF (Novoprotein, Jiangsu, China) plus 40 ng/mL RANKL (Novoprotein) for 7 days to induce human osteoclasts. The tibiae from 4-week-old C57BL/6J mice were flushed with α-MEM without FBS, and bone marrow cells were incubated in complete α-MEM for 24 h. Nonadherent cells were cultured on dishes containing M-CSF (30 ng/mL) for 3 days. The harvested adherent cells, namely bone marrow macrophages (BMMs), are used as OCPs. OCPs (8 000 cells/well) incubated in 96-well plates were treated with 30 ng/mL M-CSF (Novoprotein) and 100 ng/mL RANKL (Novoprotein) for 6 days to induce mouse osteoclasts. After osteoclastic incubation, osteoclastogenic ability was judged by Tartrate-Resistant Acid Phosphatase (TRAP) staining using related kit (BC5405, Solarbio, Beijing, China) according to manufacturer’s protocols. TRAP-positive multinucleate cells (over 3 nuclei) were considered mature osteoclasts and the size of osteoclasts were reflected by the number of large osteoclasts (over 10 nuclei).

### Western-blot assay

Cells were ultrasonically treated into lysis buffer supplemented with phosphatase and protease inhibitors (KGP2100, Keygen Biotech; Jiangsu, China). Cell lysates were transferred onto polyvinylidene fluoride membranes (PVDFs). After blockage with 5% milk, PVDFs were combined with antibodies targeting GAPDH (1:20 000, 10494-1-AP, Proteintech; IL, USA), TRAP (1:1 000, 11594-1-AP, Proteintech), CTSK (1:400, A1782, ABclonal; Wuhan, China), MMP9 (1:1 000, A0289, ABclonal), NFATC1 (1:1 000, A19597, ABclonal), CD14 (1:1 000, 17000-1-AP, Proteintech), S100A8 (1:1 000, 15792-1-AP, Proteintech), S100A9 (1:2 000, 26992-1-AP, Proteintech), S100A12 (1:1 000, A5328, ABclonal), RPLP1 (1:1 500, A6725, ABclonal), RPL13 (1:1 000, A9404, ABclonal), RPS12 (1:1 500, A5890, ABclonal), RPS28 (1:1 500, A17937, ABclonal), RPS17 (1:1 500, 16267-1-AP, Proteintech), MT-ND6 (1:2 000, A17991, ABclonal), NAMPT (1:2 000, A0256, ABclonal), CSTA (1:2 000, bsm-62286R, Bioss) and CFD (1:2 000, bs-13130R, Bioss) at 4 °C overnight. Then, PVDFs were incubated with HRP-conjugated goat anti-rabbit secondary antibody (SA00001-2, Proteintech) at 37 °C for 1 h. Finally, the signals were visualized using an Omni-ECL™Enhanced Pico Light Chemiluminescence Kit (SQ101, Epizyme Biomedical Technology, Shanghai, China) and automatic digital gel/chemiluminescence image analysis system (4600SF, Tanon, China).

### Cell immunofluorescence staining

The indicated cells (1 × 10^5^/well) were cultured on 12-well plates, fixed with 4% Paraformaldehyde (PFA), permeated, and then blocked with 1% BSA. Subsequently, the treated cells were incubated with anti-RPS17 (1:200, 16267-1-AP, Proteintech) or OSCAR (1:200, bs-17523R, Bioss) and MT-ND6 (1:200, bs-3955R, Bioss)/NAMPT (1:200, bs-0272R, Bioss)/CSTA (1:200, bs-5114R, Bioss) antibodies at 4 °C overnight. Next, cells were stained with fluorochrome-labeled secondary antibodies for 1 h and then counterstained with DAPI for 10 min. Finally, cells were observed and recorded on an inverted fluorescence microscopy (Leica DMI8, Wetzlar, German).

### Flow cytometric analysis

For flow cytometric analysis, we used MT-ND6-PE (bs-3955R, Bioss), NAMPT-PE (bs-0272R, Bioss), CSTA-PE (bsm-62286R, Bioss), CD11b-PE (340005, Biolegend), CD33-PE (303403, Biolegend) and SPARC-PE (bs-1133R, Bioss) antibodies for staining by standard protocols. Before analyzing intracellular proteins, all cells were subjected to membrane perforation.

### Coculture of CD4^+^/CD8^+^ T cells with monocytes and subsequent assay

The sorted T cells were seeded in 96-well plates coated with CD3 and CD28 antibodies (2C11, 37.51; BD Pharmingen) at 3 × 10^4^/well in proliferation medium (RPMI supplemented with 10% FBS, 5 × 10^−5^mol/L β-mercaptoethanol, sodium pyruvate and nonessential amino acids), and stained with 5 μmol/L carboxyfluorescein-succinimidyl-ester (CFSE; Elabscience, Wuhan, China). Twenty-four hours later, monocytes were added to T cells at a ratio of 1:4. Forty-eight hours later, T-cell proliferation was evaluated by flow cytometry as indicated by dilution of CFSE fluorescence.

### Electron microscopy

Cells (1 × 10^6^/sample) were prepared using regular procedures, and postfixed in 1% buffered osmium tetroxide for 2 h at 20 °C. Cell samples were dehydrated with gradient ethanol to 100%, and then infiltrated with EMbed 812 overnight. Cell samples were baked for 48 h in a 60 °C oven, and then cut into ultrathin sections (60–80 nm) using an ultramicrotome. The sections were stained with uranyl acetate, and observed on a 7700 transmission electron microscope (Hitachi, Tokyo, Japan).

### Ribosome extraction

Cells (1 × 10^7^/sample) were subjected to ribosome extraction using the corresponding Kit (BB-360632, BestBio) according to manufacturer’s protocols. Cells were centrifuged at 1 000 × *g* and 4 °C for 5 min, and the precipitation was rinsed with cold PBS, incubated with 1 mL Reagent A for 10 min on ice and homogenized for 20–30 strokes in a homogenizer. The homogenate was centrifuged at 1 000 × *g* and 4 °C for 5 min, the supernatant was transferred to a fresh tube and centrifuged at 20 000 × *g* and 4 °C for 10 min, and the harvested supernatant was centrifuged at 120 000 × *g* and 4 °C for 1 h. The collected precipitation was resuspended in 400 μL Reagent B on ice, then centrifuged at 120 000 × *g* and 4 °C for 1 h. The collected precipitation was resuspended in 250 μL Ribosome Preservation Solution, and the absorbance at 260 nm was measured using a ultramicro ultraviolet spectrophotometer (NanoDrop™ One; Thermo Fisher, Waltham, MA, USA).

### Conditional knockout mice and animal experimental protocols

RPS17^flox/flox^ (MGI:1309526, CKOCMP-20068-Rps17-B6J-VA) mice were purchased from CYAGEN (Guangzhou, China). RPS17^flox/flox^ mice were crossbred with LysM-Cre mice (No: 004781; Jackson Laboratory) to obtain RPS17-conditional knockout (CKO) mice in OCPs and flox/flox littermate control mice. Twelve-week-old female mice were subjected to bilateral ovariectomies (OVX) or sham operation under anesthesia with thiopental. Four weeks later, all mice were sacrificed. The femurs and tibiae were collected, wrapped in 0.9% saline-soaked gauze and stored at −20 °C. All mice were placed in a specific pathogen-free (SPF) facility with constant temperature and humidity on a 12-h light/dark cycling at Laboratory Animal Center of Fujian Provincial Hospital, and fed with SPF mouse feed and sterile water. All animal experiments were authorized by Institutional Animal Care and Use Committee of Fujian Provincial Hospital (IACUC-FPH-SL-20240220[0059]).

### Induction, treatment and assessment of AS-like mice

Female SKG mice were purchased from GENEANDPEACE Co., Ltd (Jiangsu China) and raised at Laboratory Animal Center of Fujian Medical University upon SPF conditions, and they underwent 12-h light/dark cycling in a temperature (22 ± 2 °C) and humidity (60%)-controlled room with free access to water and food. All mice were treated according to the guidelines for animal care approved by Institutional Animal Care and Use Committee of Fujian Provincial Hospital (IACUC-FPH-SL-20240220[0059]). AS-like alterations were induced at 10 weeks of age using 3 mg curdlan (Wako Chemicals) by intraperitoneal injection. SKG mice were randomly divided into six groups: (1) Unmodeled group treated with CD11b-promoter-Cont-AAVs; (2) CD11b-promoter-Cont-AAVs group; (3) CD11b-promoter-RPS17-AAVs group; (4) PBS group; (5) LV-Cont-infected CD14^+^CD16^−^ cells group; (6) LV-RPS17-infected CD14^+^CD16^−^ cells group. The AAV9 vectors with the monocytes/macrophages-specific CD11b-promoter carrying RPS17-cDNA or control plasmids were purchased commercially (Genechem), and AAVs were diluted to 1 × 10^13 ^μg/ml using PBS. All mice were administered with 20 μL AAVs or CD14^+^CD16^−^ cells (5 × 10^4^) via local injection into ankle-joint cavity after 1 week of induction. The two independent observers who were blinded to grouping monitored the clinical characteristics in the mice weekly. The score criteria are as mentioned previously:^43^ 0 = no swelling or redness, 0.1 = swelling or redness of the digits, 0.5 = mild swelling and/or redness of the wrist or ankle joints, and 1 = severe swelling of the larger joints. After 8 weeks of treatment, all mice were sacrificed and the ankle joints were obtained for Micro-CT scannings, hematoxylin and eosin (H&E) stainings, safranine-O/fast green (SO-FG) stainings and immunofluorescent stainings as described below. The mice in groups 2–6 were subjected to Micro-CT testing and TRAP staining for tibia tissues.

### Micro-computed tomography (Micro-CT) assay

Bruker Micro-CT Skyscan 1276 system (Kontich, Belgium) was applied for three-dimensional (3D) analysis of femur and ankle samples. Scan settings are as following: voxel size 6.5 μm, 55 kV, 200 mA, 1 mm Al filter and integration time 384 ms for cancellous bones, and voxel size 6.5 μm, 70 kV, 200 μA, 0.25 mm Al filter and integration time 350 ms for ankle samples. The reconstruction was accomplished by NRecon (version 1.7.4.2). 3D images were obtained from contoured 2D images by methods based on distance transformation of the grayscale original images (CTvox; version 3.3.0). 3D and 2D analyses were performed using software CT Analyzer (version 1.20.3.0). ZKKS-MCT-Sharp scanner (ZKKS Medical, China) was applied for 3D analysis of tibia samples from SKG modeling mice. Scan settings are as following: voxel size 10 µm, 70KV, 100 µA and integration time 100 ms. 2D and 3D images were obtained with Medproject (version 4.1) and analysis performed using software CTAn (version 1.16.8.0).

### Histology assessment

The positions of damaged/fused hip joints in AS patients were first confirmed through the preoperation imaging examinations and visual observations during surgery. Control samples were from patients with femoral head necrosis. Human femoral head samples and mouse femur and ankle-joint samples were fixed with 4% PFA for 48 h, decalcified in 20% EDTA for 30 days and then embedded in paraffin for preparing sections. The indicated sections were stained with hematoxylin for 5 min. After rinsing with PBS, eosin staining was performed for 5 min. Then, the dehydrated sections were observed and photographed using a microscopy. SO-FG staining was performed to assess human femoral heads and mouse ankle-joints. The corresponding tissues were fixed, decalcified, embedded and sectioned as described above, and then stained with 0.02% fast green fcf and 0.1% safranin O. The sections were also observed and photographed using a microscopy. TRAP staining was performed to assess mouse samples. The sections prepared as above were stained with corresponding TRAP staining kit according to manufacturer’s protocols, and the eyepiece grid was used to count the number of osteoclasts in the sections.

### Tissue immunohistochemical and immunofluorescent assays

For immunohistochemical staining, the sections from human femoral head tissues were dripped with gastric enzymes until completely covered, incubated in an oven at 37 °C for 30 min, and then removed and rinsed clean to disclose antigens. Subsequently, the sections were treated with 3% H_2_O_2_ for 25 min, blocked with 3% BSA at room temperature for 30 min, and then soaked with TRAP (1:100, GB14161, Servicebio) antibody overnight at 4 °C. The corresponding secondary antibody incubation and chromogen observation were performed using Maxvision^TM^2 HRP-Polymer anti-Mouse/Rabbit IHC Kit (MXB, Fuzhou, China; KIT-5920) according to manufacturer’s protocols. Next, the corresponding sections from human and mouse samples were subjected to immunofluorescent stainings together. The sections were deparaffinized, hydrated, and incubated in 1% Triton X-100. After incubating in 10 mM citrate buffer (pH 6.0) at 60 °C overnight to expose antigens, the sections were incubated with rabbit RANK (1:200, sc-374360, Santa Cruz Biotechnology) and RPS17 (1:200, 16267-1-AP, Proteintech) antibodies overnight at 4 °C, and then incubated with fluorescein-conjugated secondary antibodies for 1 h. Finally, DAPI was added to the sections for 10 min for counterstain. All images were obtained using a confocal fluorescence microscopy (Leica TCS SP8, Wetzlar, Germany) or Leica DMI8 fluorescence microscopy. The positive cells with double fluorescences were estimated using ImagePro Plus (IPP) 6.0 software.

### Coimmunoprecipitation (COIP) assays

COIP assays were performed using anti-RPS17 magnetic beads immunoprecipitation (IP) kit (MB202778-T46, Sino Biological; Beijing, China) by standard protocols. In brief, indicated cells were lysed using IP lysis buffer (PC105, Epizyme; Shanghai, China), and IP was conducted by incubation with magnetic beads overnight at 4 °C. The beads were rinsed three times with lysis buffer. The precipitates were eluted with loading buffer (LT101, Epizyme) and isolated for western-blotting analysis with RANK (1:500, sc-374360, Santa Cruz Biotechnology), NFATc1 (1:1 000, A19597, ABclonal), c-Fos (1:20 000, A24619, ABclonal), DCSTAMP (1:1 000, bs-8250R, Bioss) and RPS17 (1:1 500, 16267-1-AP, Proteintech) antibodies.

### Statistical analysis

The independent one-way or two-way ANOVA and Student’s *t* tests were applied to analyze the data after determining their normal distribution. Tukey test was used for Post Hoc multiple comparisons of ANOVA. All experiments were carried out using at least three independent samples. Data are represented as means ± SD. The threshold for *P* value is set to 0.05. All statistical analyses were performed using IBM SPSS 26.0 software (IBM Corp., Armonk, NY, USA).

## Supplementary information

Supplementary Figure S1-S13

Supplementary Table 1-3

## Data Availability

The scRNA-seq data are publicly available via the NCBI Sequence Read Archive (SRA) using the accession number PRJNA1168183. Other data supporting the findings in this study are available upon reasonable request.
