# Supplementary material for: Human peripheral osteoclast-precursor-development patterns reveal the significance of RPS17-dependent ribosome synthesis to Ankylosing Spondylitis lesions
Source: Bone Res. 2025 Dec 4;13:100. doi: 10.1038/s41413-025-00474-5 (PMC12678429; doi:10.1038/s41413-025-00474-5)
Supplement: Supplementary file 1 — Supplementary Figure S1-S13 [file 41413_2025_474_MOESM1_ESM.pdf]

Supplementary Figures

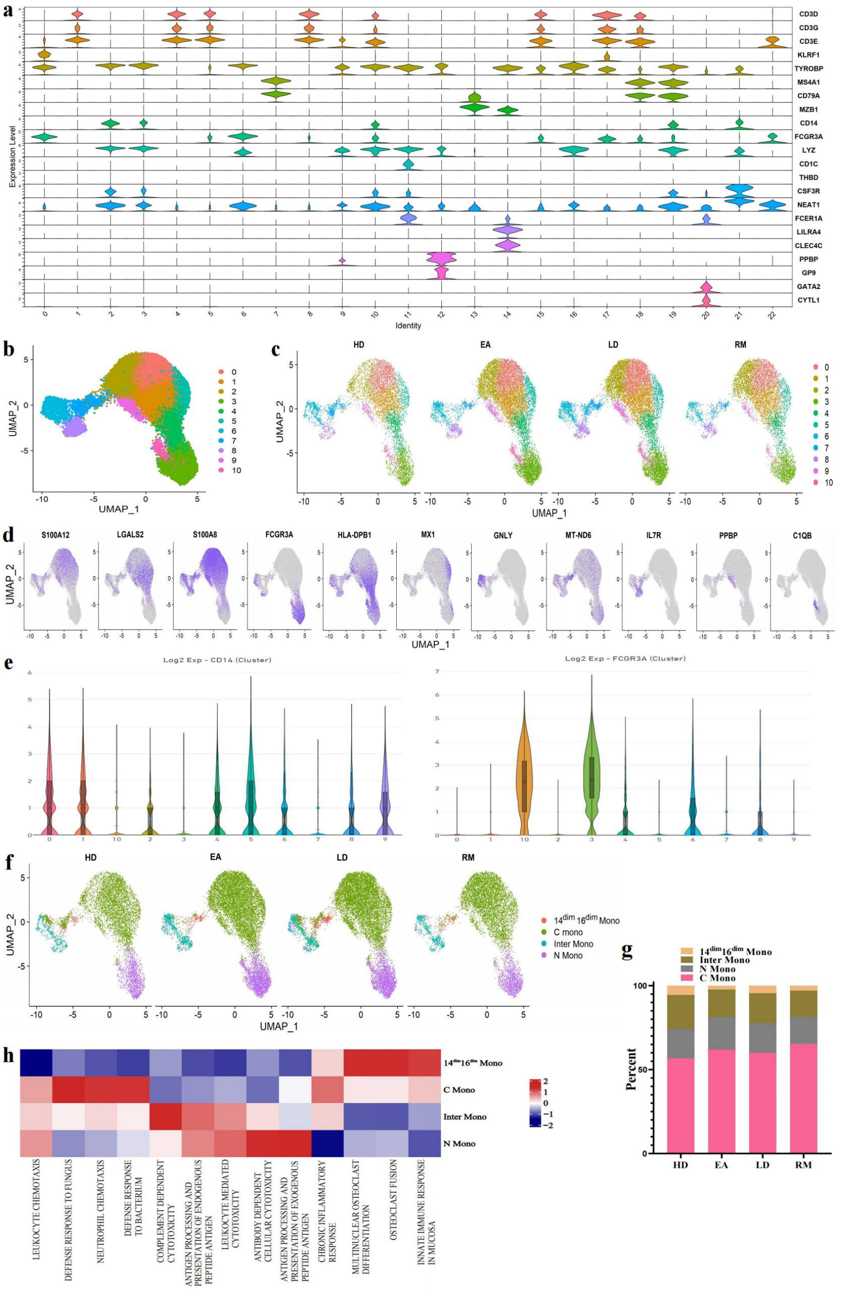

Figure S1 Supplementary data on single-monocyte transcriptional profiling. a Violin chart showing the expression distribution of typical cell markers for 23 clusters. The rows indicate various markers and the columns indicate various clusters. **b** UMAP projection of 38,654 monocytes. Each dot corresponds to a monocyte and is colored according to cell cluster. **c** UMAP projection of four conditions. 7000 cells are from HDs, 11709 cells are from EAs, 12189 cells are from LDs, and 7756 cells are from RMs. **d** Typical markers in individual UMAP plots were colored according to the expression levels and distributions. **e** Violin chart showing the expression distribution of *CD14* and *CD16*/*FCGR3A* in 11 monocyte subsets. **f** UMAP projection of four groups based on monocytic types. **g** Proportions of four subtypes from four groups. **h** The relative comparisons of GSVA scores in multiple functions between four subtypes. Columns were normalized and clustered, and the transition of blue-white-red indicates an increase in the scores.

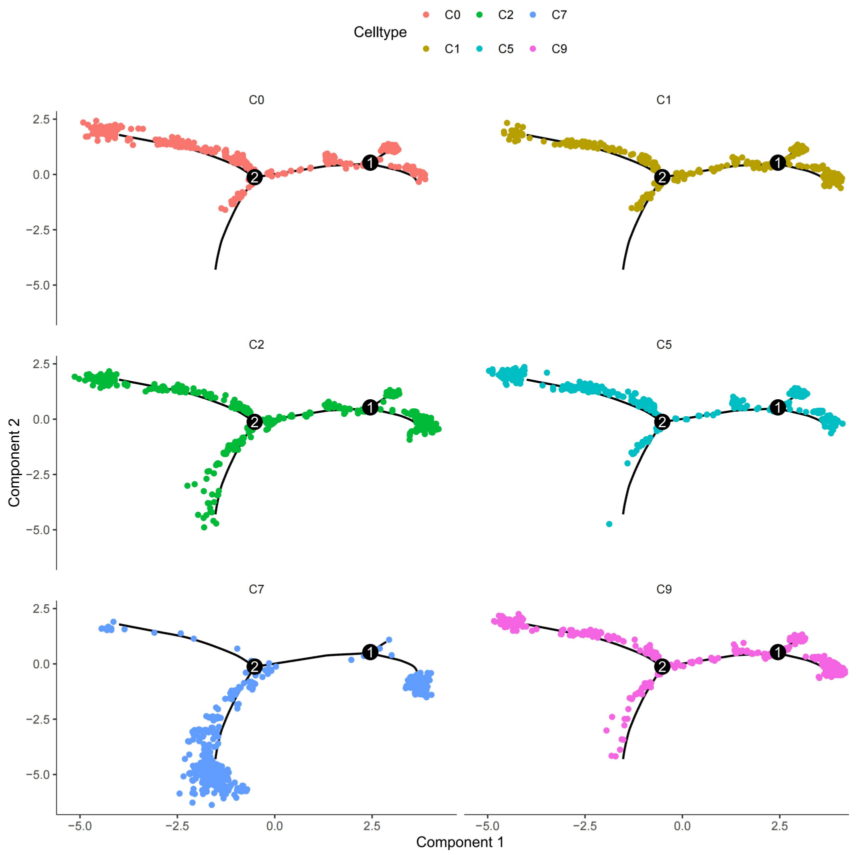

Figure S2 Supplementary data on single-cell trajectory analysis of monocytic OCPs. Monocle-2 trajectory analysis of monocyte subsets 0/1/2/5/7/9. Different dots correspond to different subsets.

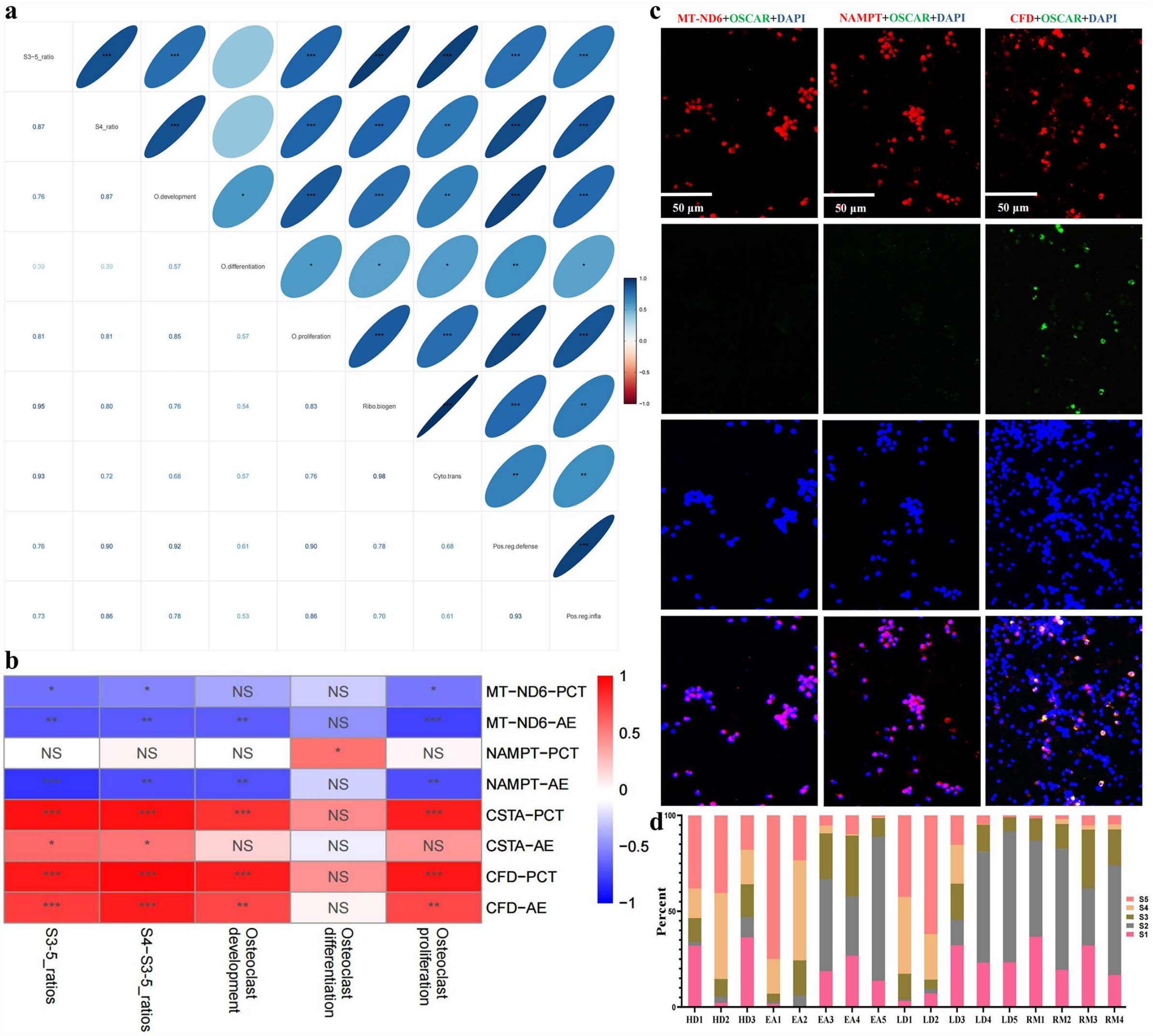

Figure S3 Supplementary data on identification of monocytic OCPs. a Correlation analysis showing that states-3/4/5 proportions and the ratios of state-4 to states-3/4/5 were positively correlated with osteoclast development/proliferation scores in the subjects, and the scores for ribosome biogenesis, cytoplasmic translation and positive regulation of defense response and inflammatory response were positively correlated with states-3/4/5 proportions, state-4—states-3/4/5 ratios, osteoclast development/differentiation/proliferation scores. **b** Correlation analysis showing that *CSTA*- or *CFD*-positive cell proportions were positively correlated with osteoclast development/proliferation scores, states-3/4/5 proportions and state-4—states-3/4/5 ratios, and *CFD* average expression was positively correlated with the four parameters while *CSTA* expression was positively correlated with states-3/4/5 proportions and state-4—states-3/4/5 ratios; nevertheless, *MT-ND6* or *NAMPT* expression was inversely correlated with osteoclast development/proliferation scores, states-3/4/5 proportions and state-4—states-3/4/5 ratios, and *MT-ND6*-positive cell proportions were inversely correlated with osteoclast proliferation scores, states-3/4/5 proportions and state-4—states-3/4/5 ratios. O.development/differentiation/proliferation, osteoclast development/differentiation/proliferation; Ribo.biogen, ribosome biogenesis; Cyto.trans, cytoplasmic translation; Pos.reg.defense/infla, positive regulation of defense response and inflammatory response. **c** Representative fluorescent images of MT-ND6/NAMPT/CFD and OSCAR for CD14^{+}CD16^{-} monocytes including single and merged fluorescences. Scale bar, 50 μm. **d** Proportions of five states at sample levels.

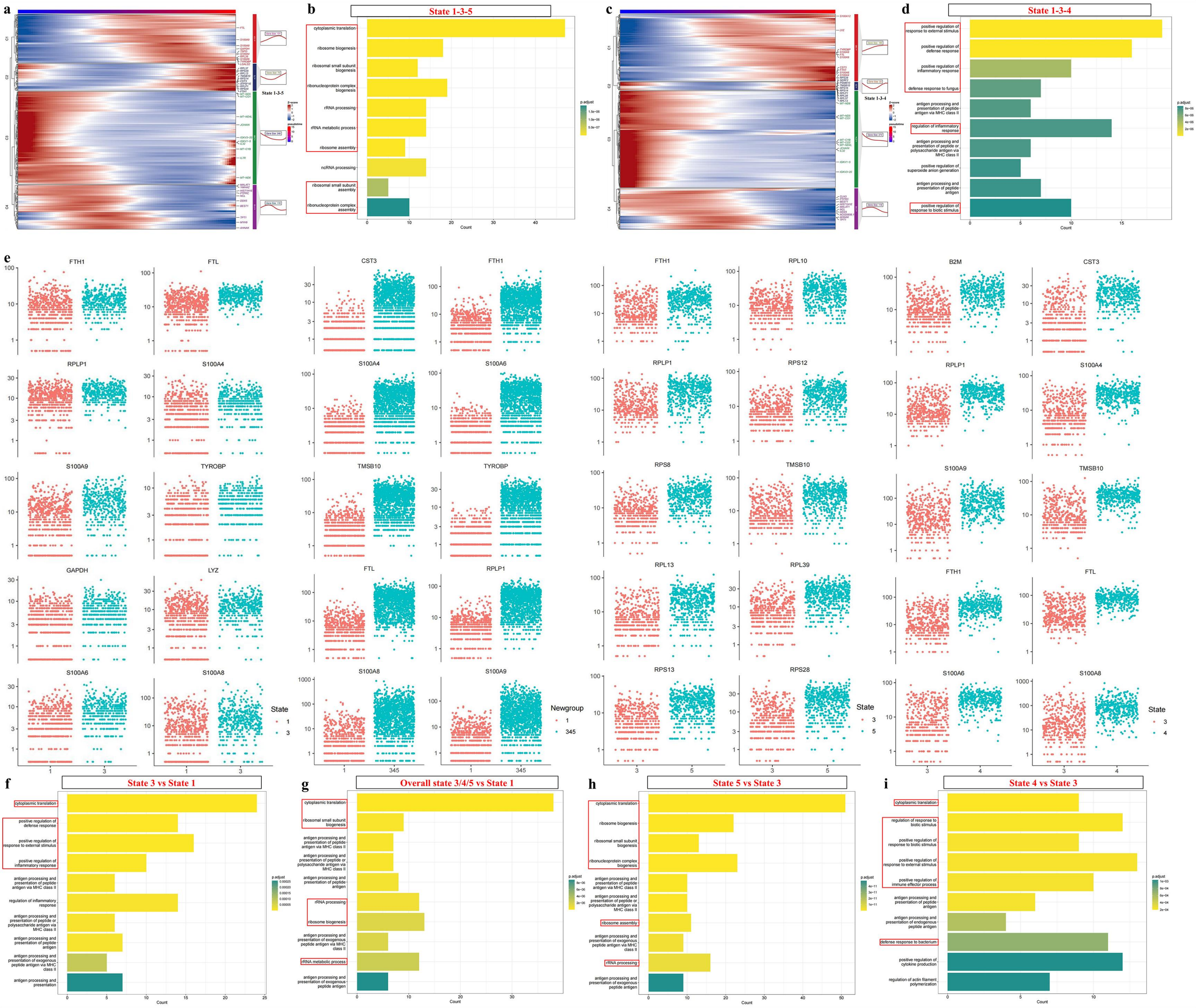

Figure S4 Supplementary data on the contribution of ribosome synthesis to peripheral OCP-development. a,c Heatmaps of four major transcription-variation clusters showing graph test of Monocle-2 pseudotime analysis for state 1-3-5 lineage and state 1-3-4 lineage, and each ten top genes are displayed on the respective clusters. **b,d** Functional enrichment of top genes for cluster-2 from dynamic analysis of state 1-3-5 lineage and cluster-1 from dynamic analysis of state 1-3-4 lineage. The top 10 GO terms are shown. The pivotal terms are marked in red box. **e** The top genes for differential analysis of cell-states between state-3 and state-1, overall state-3/4/5 and state-1, state-5 and state-3, state-4 and state-3. **f-i** Functional enrichment of top genes for differential analysis between state-3 and state-1, overall state-3/4/5 and state-1, state-5 and state-3, state-4 and state-3. The top 10 GO terms are shown. The pivotal terms are marked in red box.

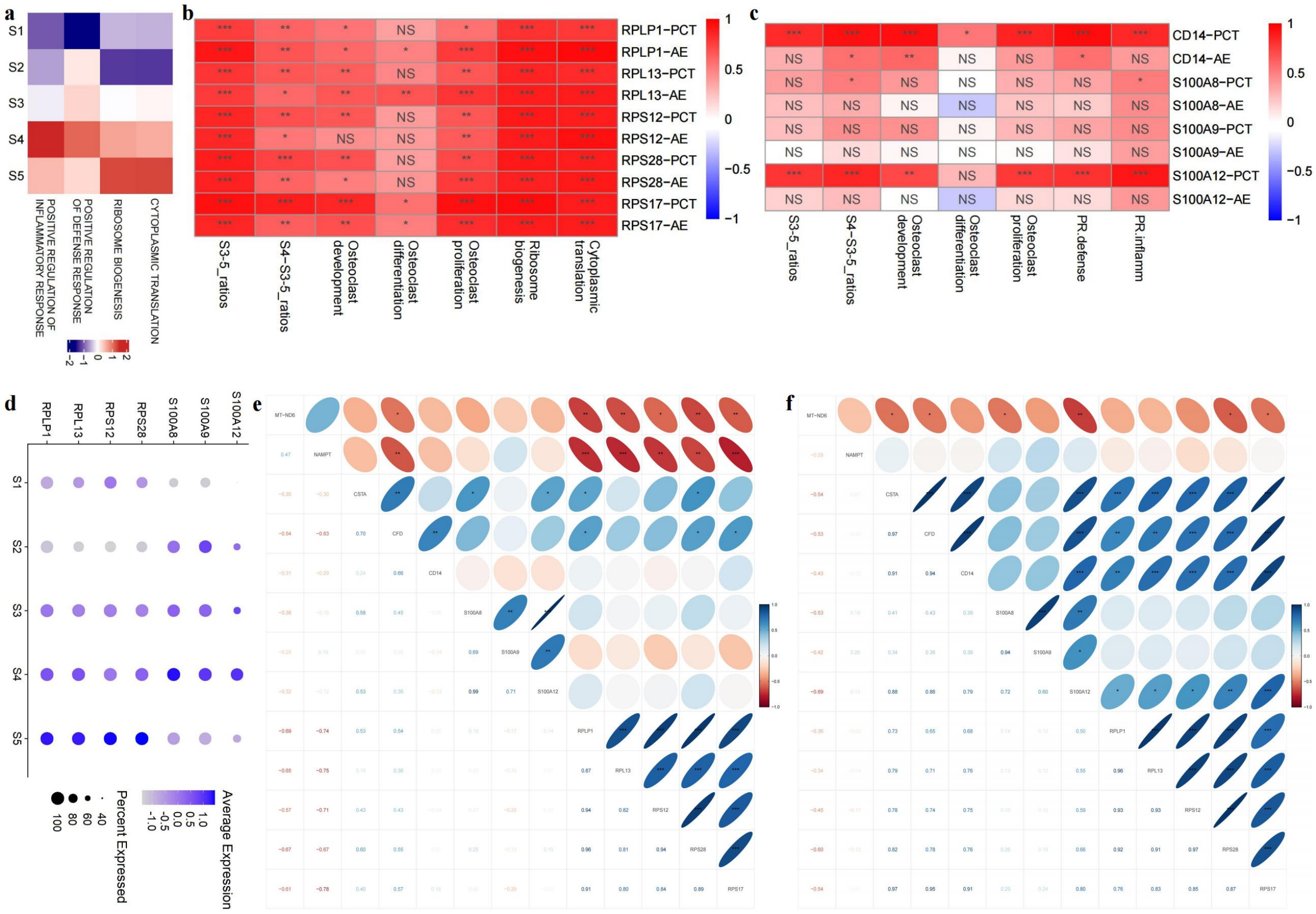

Figure S5 Correlation analysis between host-defense genes/ribosomal genes and OCP-development parameters/top state-genes. a GSVA scores in the ribosome-synthesis/host-defense functions between five states. Rows were normalized, and the transition of blue-white-red indicates an increase in the scores. **b** Correlation analysis between PCT and average levels of five major ribosomal genes and states-3/4/5 proportions, state-4—states-3/4/5 ratios, GSVA scores of osteoclast development/differentiation/proliferation, ribosome biogenesis and cytoplasmic translation. **c** Correlation analysis between PCT and average levels of four major host-defense genes and states-3/4/5 proportions, state-4—states-3/4/5 ratios, GSVA scores of osteoclast development/differentiation/proliferation and positive regulation of defense response and inflammatory response (PR.defense and PR.inflamm). **d** The expression distribution of ribosomal genes/host-defense genes across five states. The expression of RPLP1, RPL13 and RPS12/28 increased in states-3/4/5 and that of states-5 was the strongest, while the expression of *S100A8/9/12* increased in states-2-5 and that of state-4 was the strongest. **e** *RPLP1*, *RPL13* and *RPS12/28/17* average expression was inversely correlated with *MT-ND6* or *NAMPT* expression while *RPLP1*, *RPS28* and *RPS17* expression was positively correlated with *CFD* expression, and *CD14* or *S100A12* expression was positively correlated with *CFD* or *CSTA* expression respectively. **f** *MT-ND6*-positive cell proportions were inversely correlated with *RPS28*- or *RPS17*-positive cell proportions while *CSTA*- or *CFD*-positive cell proportions were positively correlated with positive cell proportions of five ribosomal molecules, and *MT-ND6*-positive cell proportions were inversely correlated with *S100A12*-positive cell proportions while *CSTA*- and *CFD*-positive cell proportions were positively correlated with *CD14*- or *S100A12*-positive cell proportions.

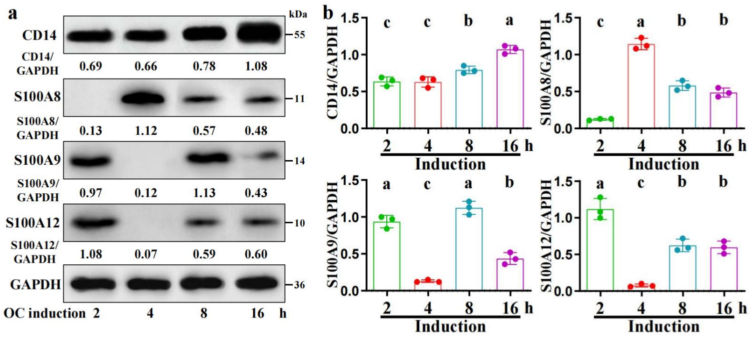

Figure S6 Western-blotting analysis of CD14, S100A8/9/12 for CD14^{+}CD16^{-} monocytes at the 2nd, 4th, 8th, and 16th hours of osteoclastic induction. The relative levels of each protein are expressed as the ratio of target protein to GAPDH. The changes from a to b/from b to c indicate a significant decrease with *P*<0.05.

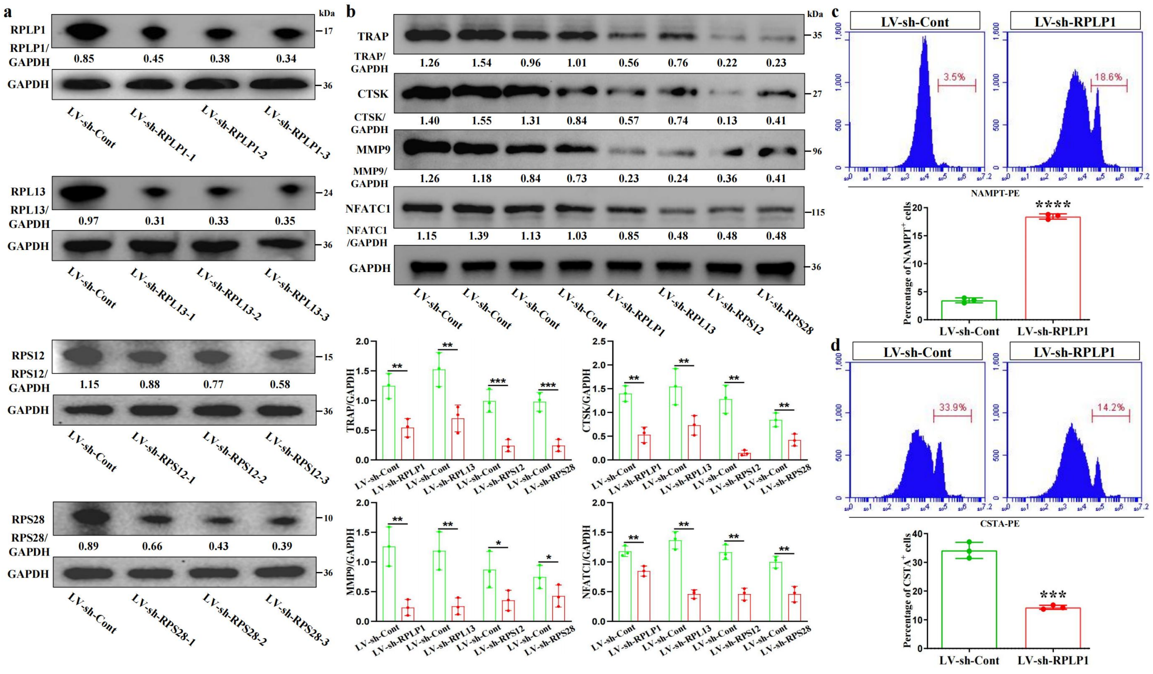

Figure S7 The impact of ribosome-molecule knockdown on OCP-development fate. a Western-blotting analysis showing the silencing efficiencies of three lentiviral vectors encoding RPLP1/RPL13/RPS12/RPS28-shRNAs. The relative levels are expressed as the ratio of RPLP1/RPL13/RPS12/RPS28 to GAPDH. According to the results, the silencing efficiency of LV-sh-RPLP1-3, LV-sh-RPL13-1, LV-sh-RPS12-3 or LV-sh-RPS28-3 was considered the strongest. Accordingly, cells transduced by the optimum shRNAs were incubated within induction medium to induce mature osteoclasts. **b** Western-blotting analysis of osteoclastic markers (TRAP, CTSK, MMP9 and NFATC1) for RPLP1, RPL13 or RPS12/28-silenced CD14^{+}CD16^{-} monocytes at the 3rd days of osteoclastic induction. Lanes 1-4 are LV-sh-Cont groups for lanes 5-8, respectively. **c-d** Flow cytometry of RPLP1-silenced CD14^{+}CD16^{-} monocytes identifying the changes in NAMPT^{+} and CSTA^{+} cells at the 16th hours of osteoclastic induction. **P*<0.05, ***P*<0.01, ****P*<0.001 by Student's t-tests (b-d).

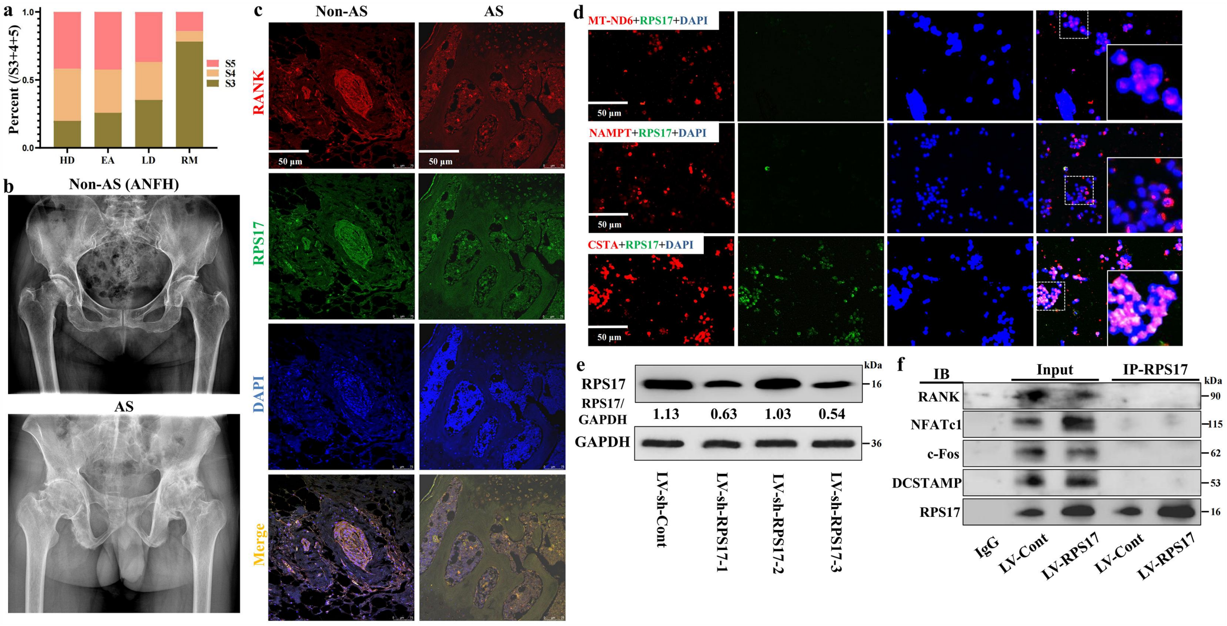

Figure S8 Supplementary data on the significance of RPS17 for monocytic OCPs from AS patients. a Respective proportion of states-3/4/5 to their total across four groups. **b** Representative images of hip-joint lesions in patients with avascular necrosis of femoral head (ANFH) and AS. **c** Representative fluorescent images of RPS17 and RANK in the hip-joint tissues including single and merged fluorescences. Scale bar, 50 µm. **d** Immunofluorescence analysis of co-expression of MT-ND6/NAMPT/CSTA and RPS17 in $CD14^{+}CD16^{-}$ monocytes. Scale bar, 50 µm. **e** Western-blotting analysis showing the silencing efficiencies of three lentiviral vectors encoding RPS17-shRNA. The relative levels are expressed as the ratio of RPS17 to GAPDH. **f** COIP analysis regarding the interaction of RPS17 and RANK/NFATc1/c-Fos/DCSTAMP in control or RPS17-overexpressed $CD14^{+}CD16^{-}$ monocytes at the 4th hours of osteoclastic induction. Data come from three repeated experiments with unanimous results. IP indicates immunoprecipitating antibody and IB indicates immunoblotting antibody.

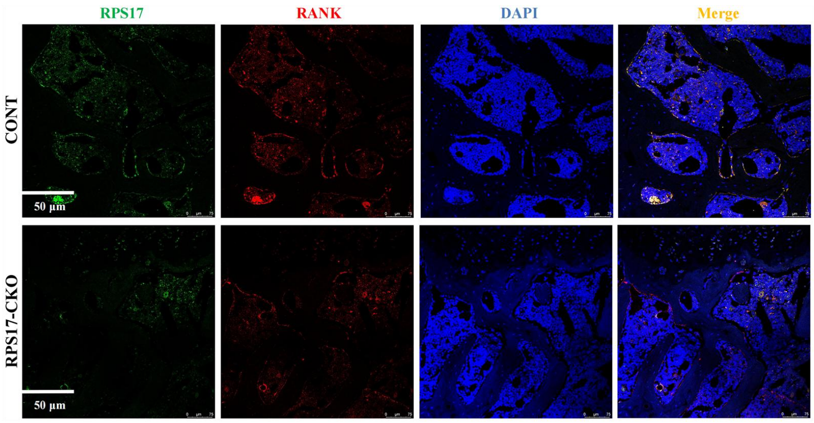

Figure S9 Supplementary data on RPS17-CKO mice. Representative fluorescent images of RPS17 and RANK in bone tissues including single and merged fluorescences. Scale bar, 50 µm.

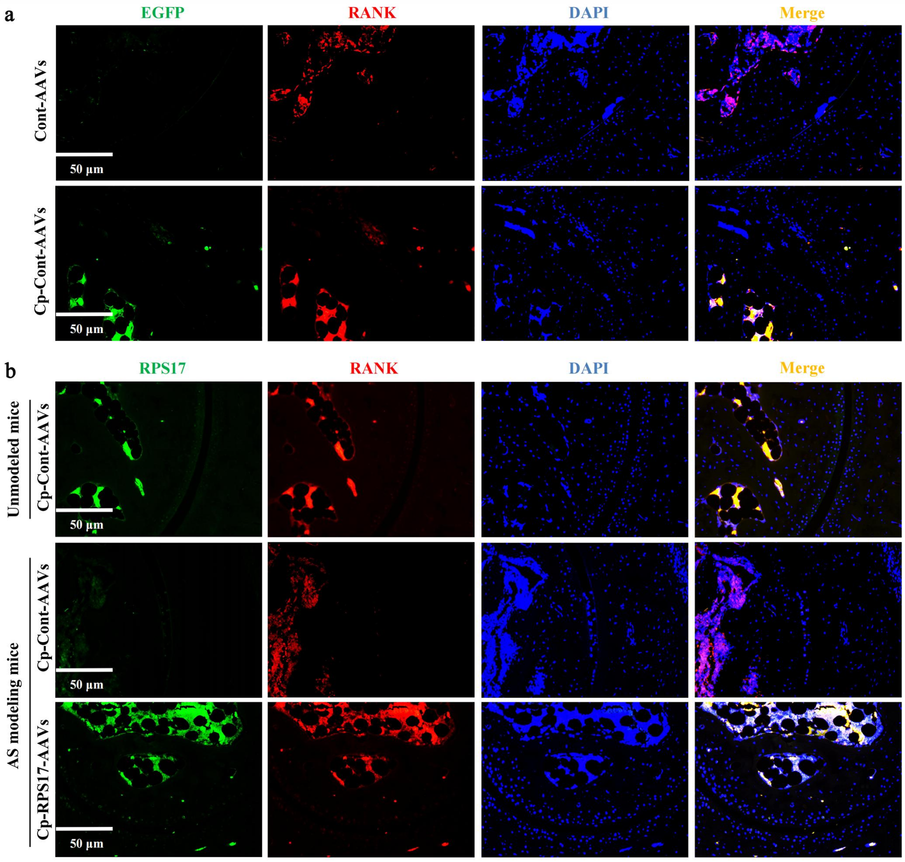

Figure S10 Supplementary data on RPS17 specific overexpression. a Representative fluorescent images of EGFP and RANK in the ankles of two groups of SKG mice including single and merged fluorescences. Scale bar, 50 μm. **b** Representative fluorescent images of RPS17 and RANK in the ankles of three groups of SKG modeling mice including single and merged fluorescences. Scale bar, 50 μm.

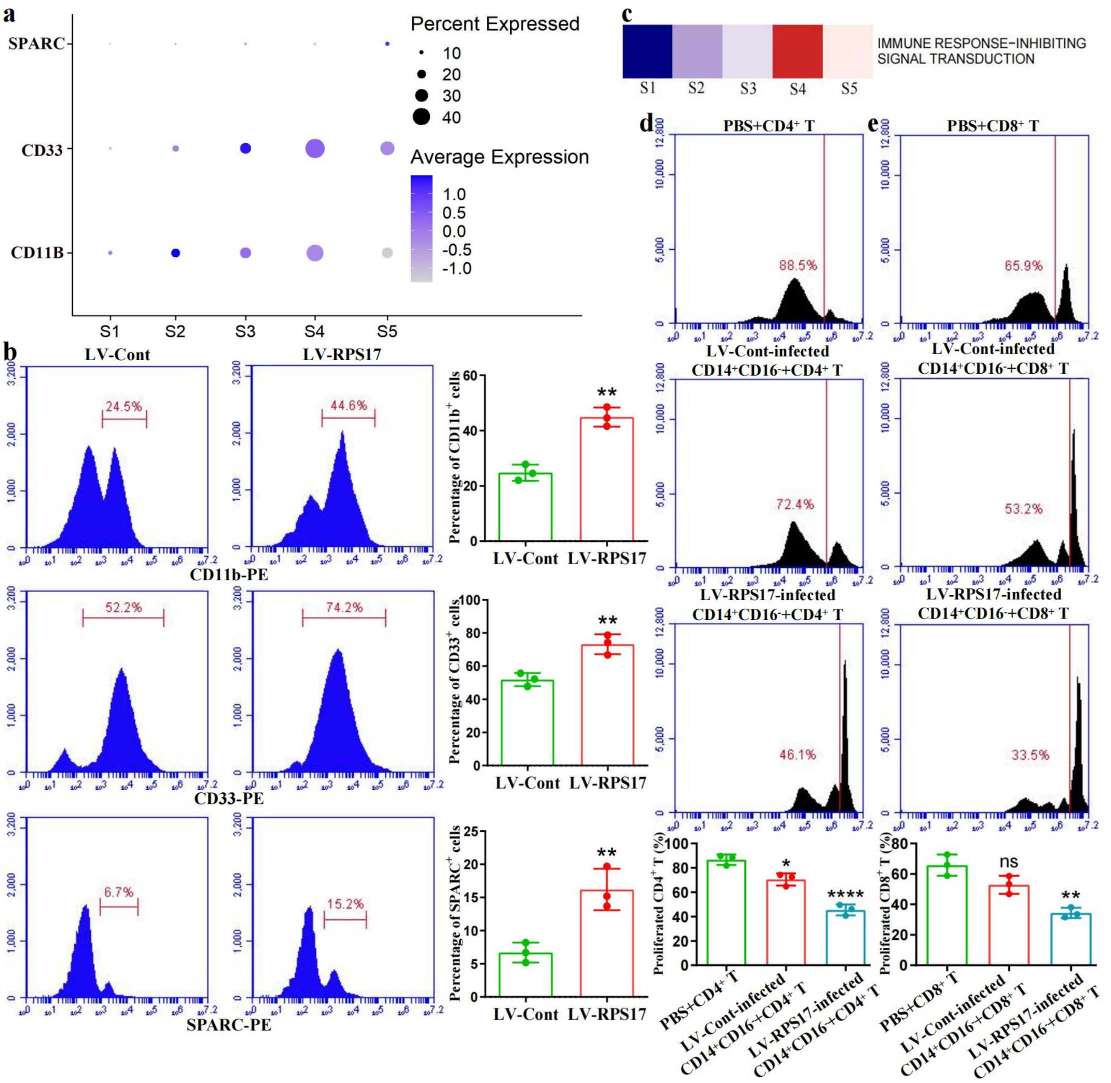

Figure S11 Attributive and functional similarities of RPS17-overexpressed monocytic OCPs to M-MDSCs. a The expression distribution of M-MDSC markers across five states. The expression of M-MDSC markers (CD11b, CD33 and SPARC) increased in states-3/4/5. **b** Flow cytometry showing that RPS17 overexpression increased the abundance of CD11b/CD33/SPARC-positive $CD14^+CD16^-$ monocytes at the 2nd hours of osteoclastic induction. **c** GSVA scores from monocyte subsets 0/1/2/5/7/9 showing that states-4/5 had the strongest capacity in suppressing immune responses. **d-e** CFSE assays showing that in the coculture system, both groups of $CD14^+CD16^-$ monocytes significantly inhibited $CD4^+$ T-cell proliferation while RPS17-overexpressed $CD14^+CD16^-$ monocytes inhibited $CD8^+$ T-cell proliferation (sorting with CD3-FITC plus CD4-APC or CD8a-APC antibodies). $^{**}P<0.01$ by Student's t-tests (b). $^{*}P<0.05$, $^{**}P<0.01$, $^{****}P<0.0001$ and ns (not significant) by One-way ANOVA (d).

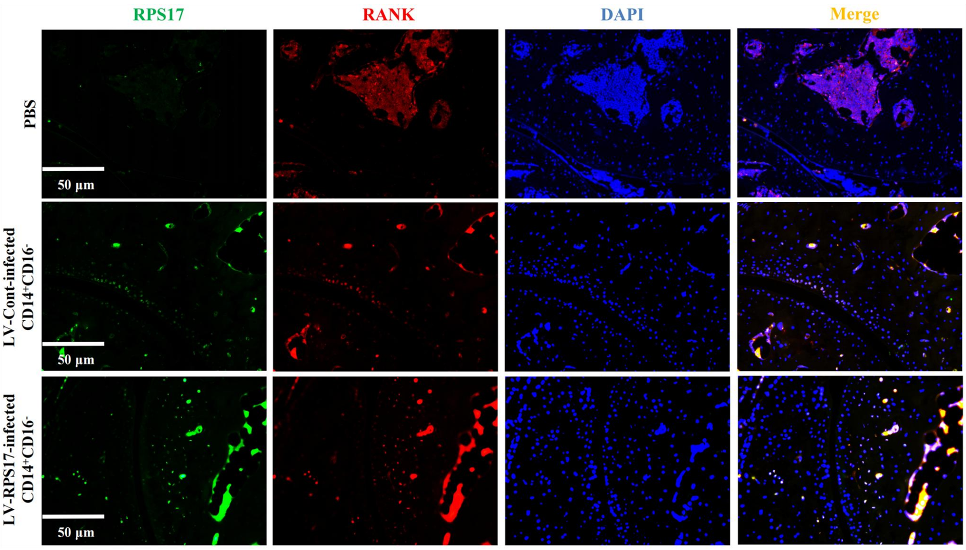

Figure S12 Supplementary data on the administration of RPS17-overexpressed monocytic OCPs. Representative fluorescent images of RPS17 and RANK in the ankles of three groups of SKG modeling mice including single and merged fluorescences. Scale bar, 50 μm.

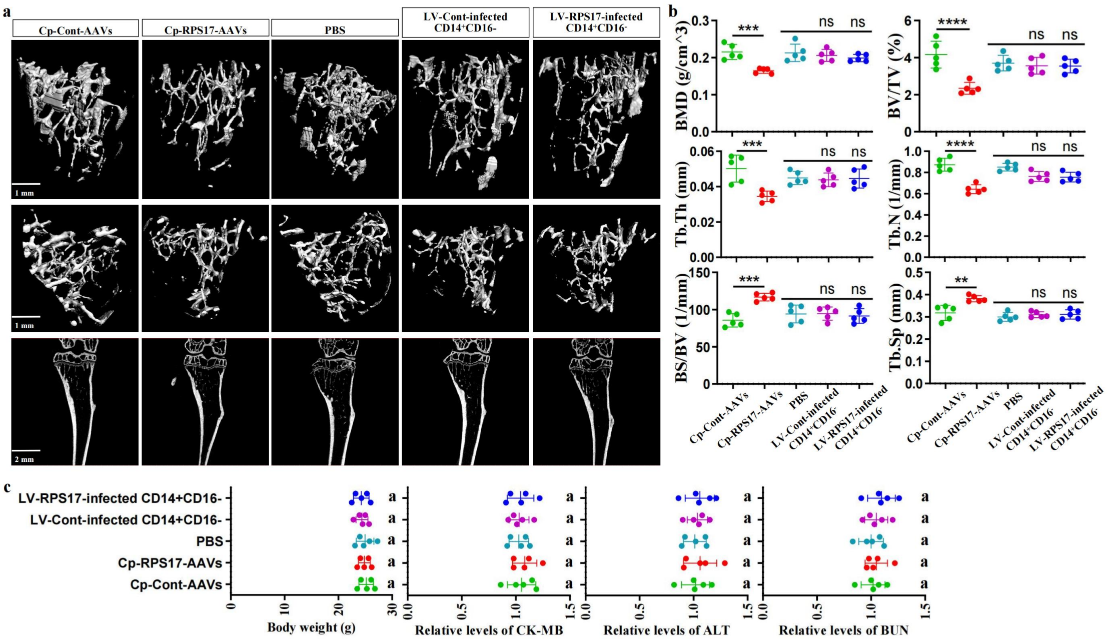

Figure S13 Safety assessment of RPS17-overexpressed monocytic OCPs. a Micro-CT showing bone mass and bone microstructure of the tibias from SKG modeling mice treated with AAVs, PBS or cytotherapies (N=5/group). **b** The trabecular bone parameters (BMD, BV/TV, Tb.Th, Tb.N, BS/BV and Tb.Sp) were analyzed using Micro-CT scanning. **c** Quantification of body weight or serum CK-MB, ALT and BUN levels. The detection of serum ALT, CK-MB and BUN relies on clinical chemistry analysis, and one sample from Cp-Cont-AAVs group was used as a control for normalization.
