# Supplementary material for: Human peripheral osteoclast-precursor-development patterns reveal the significance of RPS17-dependent ribosome synthesis to Ankylosing Spondylitis lesions
Source: Bone Res. 2025 Dec 4;13:100. doi: 10.1038/s41413-025-00474-5 (PMC12678429; doi:10.1038/s41413-025-00474-5)
Supplement: Supplementary file 2 — Supplementary Table 1-3 [file 41413_2025_474_MOESM2_ESM.pdf]

Supplementary Table 1 Characteristics of the subjects regarding scRNA-seq

	HD	EA	LD	RM
Number	3	5	5	4
Age, year	32.67±2.08	33.8±0.84	38.8±3.11	33.75±8.34
Number (%) male	3 (100%)	4 (80%)	4 (80%)	3 (75%)
Number (%) HLA-B27^{+}	3 (100%)	5 (100%)	5 (100%)	4 (100%)
Disease duration, month	NA	2.2±0.57^{c}	89.6±26.79^{a}	27.25±8.1^{b}
Remission duration, month	NA	0^{b}	0^{b}	17.25±12.5^{a}
CRP, mg/L	NA	4.66±2.82^{b}	16.34±10.53^{a}	0.81±0.1^{c}
ESR, mm/h	NA	14±7.31^{b}	35.8±19.42^{a}	2.5±1.73^{c}
BASDAI	NA	5.88±1.71^{a}	5.12±1.8^{a}	0.65±0.3^{b}
BASFI	NA	5.16±2.35^{a}	7.68±1.46^{a}	1.35±0.53^{b}

Mean ± SD. HD, healthy donors; EA, patients in early-active stage; LD, patients in late-dysfunction stage; RM, patients in clinical remission; HLA-B27, human leukocyte antigen B27; CRP, C-reactive protein; ESR, Erythrocyte sedimentation rate; BASDAI, Bath ankylosing spondylitis disease activity index; BASFI, Bath ankylosing spondylitis functional index; NA, Not Applicable. The demotion in letters indicates a significant decrease with P<0.05.

Supplementary Table 2 Characteristics of the subjects for histology assessment

	**Non-AS**	**AS**
Number	6	6
Age, year	46.17±6.01	37.5±5.13
Number (%) male	5 (83%)	6 (100%)
Number (%) HLA-B27^{+}	0	6 (100%)
Disease duration, month	NA	56.33±16.31
CRP, mg/L	3.13±1.62	18.81±2.85
ESR, mm/h	10.33±7.31	38.33±6.77
BASDAI	NA	5.27±0.61
BASFI	NA	6.43±1.59

Mean ± SD. AS, ankylosing spondylitis; HLA-B27, human leukocyte antigen B27; CRP, C-reactive protein; ESR, Erythrocyte sedimentation rate; BASDAI, Bath ankylosing spondylitis disease activity index; BASFI, Bath ankylosing spondylitis functional index; NA, Not Applicable.

Supplementary Table 3 Characteristics of the additional subjects for blood testing

	HD	EA	LD	RM
Number	6	6	6	6
Age, year	31.5±5.17	28.5±4.37	39.17±4.02	32.5±3.78
Number (%) male	5 (83%)	5 (83%)	5 (83%)	5 (83%)
Number (%) HLA-B27$^+$	0	6 (100%)	6 (100%)	6 (100%)
Disease duration, month	NA	2.08±0.58^c	81.33±39.57^a	31.17±19.92^b
Remission duration, month	NA	0^b	0^b	18.83±17.15^a
CRP, mg/L	NA	3.81±0.93^b	12.42±3.37^a	1.49±0.93^c
ESR, mm/h	NA	13.83±2.48^b	30.83±7.63^a	7.17±4.17^c
BASDAI	NA	4.87±0.97^a	5.07±0.79^a	0.82±0.33^b
BASFI	NA	3.25±1.09^b	7.47±1.67^a	1.75±0.68^c

Mean ± SD. HD, healthy donors; EA, patients in early active stage; LD, patients in late dysfunction stage; RM, patients in clinical remission; HLA-B27, human leukocyte antigen B27; CRP, C-reactive protein; ESR, Erythrocyte sedimentation rate; BASDAI, Bath ankylosing spondylitis disease activity index; BASFI, Bath ankylosing spondylitis functional index; NA, Not Applicable. The demotion in letters indicates a significant decrease with $P<0.05$.
